# Evidence to Support the Collaboration of SP1, MYC, and HIF1A and Their Association with microRNAs

**DOI:** 10.3390/cimb46110741

**Published:** 2024-11-05

**Authors:** Jong Ho Chun, Kotohiko Kimura, Monika Rajput, Ming-Hua Hsu, Yu-Chuan Liang, Akanksha Ramadas Shanbhag, Pei-Ju Chiang, Tiffany L. B. Jackson, Ru Chih C. Huang

**Affiliations:** 1 Department of Biology, Johns Hopkins University, 3400 N. Charles St-Levi Hall 250, Baltimore, MD 21218, USAmonika05rajput@gmail.com (M.R.); ashanbh1@jhu.edu (A.R.S.);; 2 Department of Surgical Oncology, Institute of Medical Sciences, Banaras Hindu University, Varanasi 221005, Uttar Pradesh, India; 3 Department of Chemistry, National Changhua University of Education, Changhua 500, Taiwan; 4 Agricultural Biotechnology Research Center, Academia Sinica, Taipei 11529, Taiwan; 5 Advanced Academic Programs, Johns Hopkins University, Baltimore, MD 21218, USA; 6 Department of Biomedical Engineering, Johns Hopkins University, Baltimore, MD 21218, USA; pchian13@jh.edu; 7 Academia Sinica, Taipei 115, Taiwan

**Keywords:** M_4_N, SP1, MYC, HIF1A, anticancer, stem cell

## Abstract

This study provides evidence to support the concept proposed by Kimura et al. in 2023 that the inhibitors of SP1, MYC, and HIF1A should induce strong anticancer activity by reducing the expression of stem cell-related proteins. In LN229 and U87MG glioblastoma cells, either tetra-methyl-O-nordihydroguaiaretic acid (M_4_N) or tetra-acetyl-O-nordihydroguaiaretic acid (A_4_N) suppressed SP1 and only a few stem cell-related proteins and induced only a small amount of cell death; in contrast, the combination treatment of M_4_N with A_4_N greatly suppressed the expression of SP1, MYC, and HIF1A, as well as all of the stem cell-related proteins examined, and greatly induced cell death. The bioinformatic analysis showed that the proteins associated with SP1, MYC, and HIF1A were specifically involved in the regulation of transcription and that various microRNAs (miRNAs) that had been shown to induce either anti- or procancer activity were associated with SP1, MYC, and HIF1A, which suggested that the inhibition of SP1, MYC, and HIF1A could modulate the transcription of both coding and noncoding RNAs and affect cancers. These data overall supported our concept.

## 1. Introduction

Tetramethyl-O-nordihydroguaiaretic acid (Terameprocol; M_4_N) is an anticancer drug candidate that has been studied for many years [1,2,3,4,5,6]. M_4_N is a derivative of nordihydroguaiaretic acid (NDGA), a lignan, which was first discovered in the creosote bush (Larrea divaricata), a desert medicinal plant. The anticancer efficacy of M_4_N was first shown in a clinical trial for oral cancer patients conducted in India two decades ago [7,8]. The results of a recent phase I clinical trial for glioblastoma (GBM) patients conducted by multiple research institutions in the United State are also available [9]. This compound reversibly inhibits the binding of SP1, a major transcription factor, to its DNA consensus sequence, thereby functioning as an SP1 inhibitor unlike mithramycin, which is an irreversible inhibitor of SP1 [5]. M_4_N also decreases the cellular content of HIF1A, another major transcription factor, in cancer cells under hypoxia, indicating that it functions as a dual inhibitor of both SP1 and HIF1A [10]. One of the most important features of M_4_N is that it essentially has no strong side effects [11], suggesting that the reversible inhibition of SP1 and HIF1A binding to their target genes might not cause strong toxicity. SP1, a constitutively expressed housekeeping gene, regulates diverse yet distinct biological activities, while HIF-1, whose protein level is rapidly increased when the local tissue oxygen concentration decreases, functions as a mediator of hypoxic signals. Both are very important transcription factors that regulate a great number of genes. Therefore, M_4_N is expected to affect diverse biological mechanisms, causing a strong negative impact on cancer [10,12,13,14]. This nature of M_4_N to possess multiple activities is useful in dealing with cancers, which are inherently composed of various cancer cells with a diverse range of gene mutations.

In addition to the anticancer activity of M_4_N as a single drug, it has been shown by many mouse xenograft studies that M_4_N has activity to induce anticancer activity synergistically with a second anticancer drug of various types as well [10,13]. This is probably because M_4_N induces a negative impact on cancers by its activity that influences a broad range of biological processes as a dual inhibitor of SP1 and HIF1A and makes cancers susceptible to a second anticancer drug. Meanwhile, it was shown by several systems analyses that SP1, HIF1A, and MYC functioned as master regulators of cancer development [15]. Since M_4_N is a dual inhibitor of SP1 and HIF1A, we hypothesized that M_4_N should induce strong anticancer activity with a second anticancer drug that functions as a MYC inhibitor [15]. In addition, it was shown by promoter analysis that the expression of many cancer stem cell (CSC)-related genes such as TERT, BMI1, BSG (CD147), and PROM1 (CD133) was under the control of SP1, HIF1A, and MYC [15]. It has been shown that CSCs, although they are among the smallest population in most cancers, play pivotal roles in the pathogenesis and progression of cancers including blood cancers and solid tumors [16]. Therefore, if the combination treatment of SP1, MYC, and HIF1A inhibitors can suppress the expression of CSC-related genes, this activity should be a crucial point in the mechanism of how the combination treatment works.

In this study, we introduced a new NDGA derivative, tetra-acetyl-O-nordihydroguaiaretic acid (A_4_N), which has more hydrophilic characteristics than M_4_N and can be dissolved in water more easily than M_4_N, and examined the effect of M_4_N and A_4_N treatment on the expression of SP1, MYC, HIF1A, and various proteins related to CSCs and epithelial–mesenchymal (EM) transition (which was strongly related to CSCs) [17,18], as well as on cell death induction in LN229 and U87MG GBM cells. In addition, bioinformatics analyses regarding SP1, MYC, and HIF1A were performed to explore the theoretical basis of our concept about an anticancer therapy based on SP1, MYC, and HIF1A inhibitors in combination.

## 2. Materials and Methods

### 2.1. Reagents

M_4_N was supplied by Erimos Pharmaceutical, LLC (Raleigh, NC, USA). Anti-SP1 (CST9389), anti-c-MYC (CST 9402), anti-PROM1 (CST 86781), anti-BMI-1 (CST 5855), anti-SOX2 (CST 2748), anti-SNAIL (CST 3879), anti-TWIST (CST 90445), and anti-ZEB1 (CST 70512) antibodies were from Cell Signaling Technology (Danvers, MA, USA). Anti-HIFA1 (PA5-85494) and anti-TERT (PA5-86750) antibodies were from Invitrogen (Carlsbad, CA, USA). Other reagents were purchased from Millipore Sigma (St. Louis, MO, USA).

### 2.2. Production of Tetra-O-acetoxyl-nordihydroguaiaretic Acid (A_4_N)

NDGA 1 g was heated in acetic anhydride (10 mL) at 80 °C for 12 h. After diluting with water, the mixture was neutralized with saturated Na_2_CO_3_ solution and extracted with dichloromethane. The organic layer was dried with anhydrous MgSO_4_, and the solvent was evaporated under reduced pressure. Recrystallization (MeOH) was performed twice at 0 °C to obtain the product (white solid).

### 2.3. Cell Culture

LN229 and U87MG human GBM cells were purchased from American Type Culture Collection (Manassas, VA, USA) and were maintained in DMEM supplemented with FBS (5%) at 37 °C.

### 2.4. Western Blot Analyses

Western blotting was performed as previously described [10]. After the cells had been grown in 25 mm^2^ flasks and treated with reagents, they were washed with phosphate-buffered saline (PBS; 137 mM NaCl, 2.7 mM KCl, Na_2_HPO_4_ 10 mM, and KH_2_PO_4_ 1.8 mM) three times and suspended in RIPA buffer (150 mM NaCl, 50 mM Tris-HCl [pH 8.0], 0.1% SDS, 1% NP40, and 0.5% deoxycholate) supplemented with protease inhibitor cocktail (Calbiochem, San Diego, CA, USA). The viscous cell lysate was sonicated for 5–10 s with a Vibra-Cell sonicator (Sonics and Materials, Newtown, CT, USA) with the microtip set at 40% output. The sample volumes were adjusted by the total protein amount. The protein assay was performed with the Bio-Rad Protein Assay Kit (Bio-Rad Laboratories Inc., Hercules, CA, USA). The samples were resolved by standard SDS–polyacrylamide gel electrophoresis and transferred to Hybond-ECL nitrocellulose membrane (Amersham Biosciences, Bjorkgatan, Sweden) using a semi-dry electroblot apparatus. The membranes were blocked with skim milk and incubated with primary antibodies at 4 °C overnight and then with secondary antibody conjugated with horseradish peroxidase at room temperature for 2 h. The signals were detected by Western blot chemiluminescence reagent plus (New England Nuclear Life Science Products, Boston, MA, USA). The antibodies are listed in the supplement.

### 2.5. Cell Death Analyses

Flow cytometry was conducted using a BD FACSCanto™ II system. LN229 and U87MG cells were harvested via trypsinization with 0.25% trypsin–EDTA, washed with 1× PBS, and centrifuged at 300× *g* for 5 min. After washing with 1× PBS and centrifugation, the cells were subjected to PE Annexin V (#640908, Biolegend, San Diego, CA, USA) and 7-Aminoactinomycin D (7-AAD; #79993, Biolegend) staining according to the manufacturer’s guidelines. Following a final wash, the cells were resuspended in staining buffer (0.5% BSA in PBS) and analyzed on the flow cytometer. The statistical analyses were performed using Student’s *t*-test (SigmaPlot; SPSS Inc., Chicago, IL, USA).

### 2.6. Bioinformatics Analysis

Biological Network integration: To better understand how the key proteins in our study interact with other proteins, we used the NetworkAnalyst tool (available at http://www.networkanalyst.ca) (accessed on 26 March 2024) [19]. NetworkAnalyst helps us visualize and analyze complex relationships between proteins. The protein–protein interaction (PPI) network was constructed based on the STRING interactome database (https://string-db.org) (accessed on 13 August 2024) [20]. With SP1, MYC, and HIF1A as our key proteins, we constructed an expanded protein interaction network. Additionally, Gene–miRNA interaction networks were constructed for the SP1, MYC, and HIF1A genes by leveraging the miRTarBase v8.0 database (https://mirtarbase.cuhk.edu.cn/) (accessed on 4 June 2024), a curated repository of experimentally validated miRNA–target interactions through the NetworkAnalyst tool [21]. Further analysis of the involved genes was carried out by constructing a gene–gene interaction (GGI) network using the GeneMania online tool (https://genemania.org/) (accessed on 27 March 2024) [22].

Gene Ontology: To gain deeper insights into the biological functions of the identified genes, we performed Gene Ontology (GO) enrichment analysis. We performed the GO analysis by using the Gene Set Enrichment Analysis (GSEA) online bioinformatics tool (https://www.gsea-msigdb.org/gsea) (accessed on 06 August 2024) [23]. This approach allows for the functional annotation and prediction of biological roles based on standardized gene-associated terms.

Pathway analysis: To perform REACTOME Pathway analyses, the WebGestalt tool (WEB-based Gene SeT AnaLysis Toolkit) was utilized. WebGestalt is an online bioinformatics platform designed for functional enrichment analysis, enabling researchers to explore significantly enriched genes and functional pathways (http://www.webgestalt.org) (accessed on 13 August 2024) [24]. This tool supports various types of analyses, including Over Representation Analysis (ORA), GSEA, and Network Topology-based Analysis (NTA), and is compatible with multiple organisms and gene identifiers. In this study, WebGestalt was employed to identify enriched pathways and gene sets, leveraging its capabilities to provide insights into the biological significance of the gene lists under investigation.

Disease Ontology Analysis: To further elucidate the disease associations of the identified genes, we performed Disease Ontology (DO) analysis using the WebGestalt tool (http://www.webgestalt.org) (accessed on 13 August 2024) [25]. This approach allowed for the systematic exploration of disease-related biological processes and pathways potentially implicated in our study.

## 3. Results

### 3.1. The Introduction of a New NDGA Derivative, Tetra-O-acetoxyl-nordihydroguaiaretic Acid (A_4_N)

**Figure 1:**
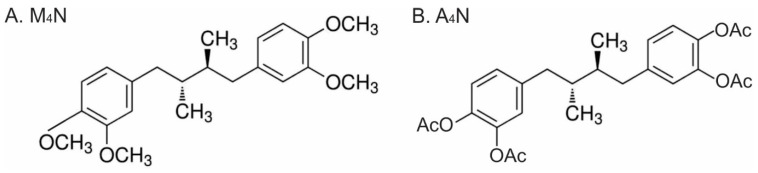
A comparison of the chemical structures of M_4_N and A_4_N. The chemical structures of two NDGA derivatives, M_4_N (**A**) and A_4_N (**B**), are shown. AcO means an acetoxyl group.

The NDGA derivative M_4_N has been studied for many years as an anti-carcinogenic and an anti-viral compound [1,2,3,4,5,6]. Although it was shown by numerous experiments that M_4_N had anti-carcinogenic and anti-viral activities, one major shortcoming of M_4_N was that it is hard to dissolve in aqueous solutions including serum since M_4_N is a rather lipophilic (hydrophobic) compound. Due to this chemical property of M_4_N, it is difficult to increase the serum concentration of this compound after its administration into either animals or humans, and it is necessary to administer M_4_N for many weeks to achieve a high concentration of this drug in the target organs. To resolve this shortcoming, a new NDGA derivative, tetra-O-acetoxyl-nordihydroguaiaretic acid (A_4_N), was synthesized (Figure 1). Due to the four acetoxyl groups that replace the four methyl groups in the M_4_N molecule, A_4_N is by far more hydrophilic than M_4_N, and in fact, A_4_N can be dissolved in water easily, unlike M_4_N, which requires a special solvent to be dissolved. One great advantage of M_4_N is that this compound is extremely non-toxic. The data so far indicated that A_4_N was just as non-toxic as M_4_N.

### 3.2. Either M_4_N or A_4_N Suppresses the Expression of SP1, HIF1A, MYC, CSC-Related Proteins, and EM Transition-Related Proteins in LN229 and U87MG GBM Cell Lines

Previously, we explored the concept that SP1, HIF1A, and MYC collaborate together to promote cancer development, based on comprehensive investigations of various system analyses of cancer-related genes and promoter analyses of SP1, MYC, and HIF1A, and that the inhibition of the expression of all of these transcription factors might be a key to suppressing cancer development [15]. We also examined whether the suppression of SP1, MYC, and HIF1A could reduce the expression of various stem cell-related genes that played important roles in cancer development, which suggested the possibility that M_4_N, as an SP1/HIF inhibitor that lacks activity to inhibit MYC, might induce anticancer activity via its effect on suppressing the expression of stem cell-related genes to some extent [15]. It is known that there are numerous stem cell-related proteins such as TERT, BMI1, and PROM1 [15]. Among them, PROM1 is the most well known since PROM1, a penta-span membrane surface protein, is often used as a CSC marker [26]. It was shown that PROM1-positive GBM cells exhibited stem cell-related traits such as self-renewal, multipotency, and high proliferation potential [27,28] and that as few as one hundred PROM1-positive cells were capable of initiating tumors in immunodeficient mice that closely resembled the original tumors of patients, thus highlighting the remarkable tumorigenicity of these PROM1-positive tumor cells [29]. PROM1 is also notably expressed at high levels in various GBM cell lines. For instance, LN229 and U87MG cells demonstrate the formation of neurosphere-like cell aggregates indicative of stem cell characteristics upon prolonged culture and exhibit the properties of self-renewal, differentiation, and tumorigenicity [30,31]. Meanwhile, the EM transition is often considered to be associated with de-differentiation. Since mature cells are produced from stem cells by differentiation, EM transition is indicative of reversing differentiation to generate stem cells from differentiated mature cells [32].

**Figure 2:**
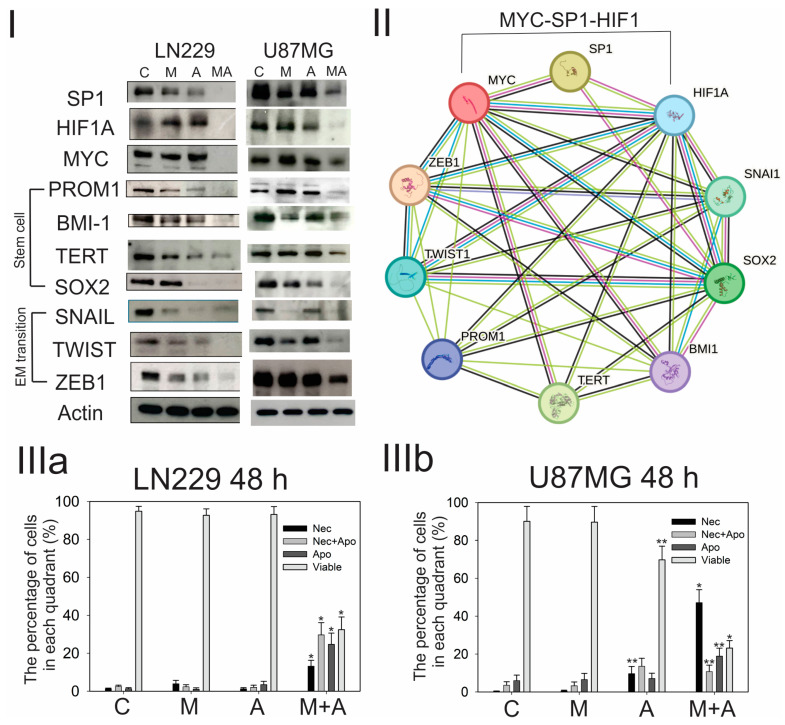
The effect of M_4_N and A_4_N on the expression of SP1, MYC, HIF1A, and proteins associated with stem cells and EM transition. (**I**) Western blotting to show the effect of M_4_N and A_4_N on these proteins in LN229 and U87MG GBM cells. Either LN229 or U87MG cells were treated with M_4_N (60 μM) and/or A_4_N (60 μM) for 48 h, and the content of each protein was examined by Western blotting. C: Control, M: M_4_N (60 μM), A: A_4_N (60 μM), and MA: M_4_N (60 μM) and A_4_N (60 μM) combination treatment. The full blotting images are in the supplement (Supplementary Figure S2). (**II**) Construction of the PPI network through the STRING database for the validation of the associations among these proteins. A PPI network was constructed with 10 nodes (SP1, HIF1A, MYC, PROM1, BMI-1, TERT, SOX2, SNAIL, TWIST, and ZEB1). SNAI1 is an alias of SNAIL. Known Interactions: curated databases—light blue; experimentally determined—pink; Predicted Interactions: gene neighborhood—green; gene fusions—red; gene co-occurrence—dark blue; Others: Text-mining—yellow; co-expression—black; protein homology—turquoise. (**III**) Cell death induced by M_4_N and A_4_N in LN229 (**a**) and U87MG (**b**) cells. Cell death was examined at 48 h after treatment. C: Control, M: M_4_N (60 μM), A: A_4_N (60 μM), and M+A: M_4_N (60 μM) and A_4_N (60 μM) combination treatment. Cell death was assayed by the fluorescence-activated cell sorting (FACS)-based analysis using Annexin V as an indicator for apoptosis (Apo) and 7-AAD staining as an indicator for necrosis (Nec). In the category column, ‘Nec’ indicates the percentage of necrotic cells by the Annexin-positive and 7-AAD-negative staining, ‘Nec+Apo’ indicates both necrotic and apoptotic cells by the Annexin-positive and 7-AAD-positive staining, ‘Apo’ indicates apoptotic cells by the Annexin-positive and 7-AAD-negative staining, and ‘Viable’ indicates the viable cells by the Annexin-negative and 7-AAD-negative staining. There were statistically significant differences in the percentage of cells in each quadrant between C and M+A in both LN229 and U87MG cells by Student’s *t*-test (* indicates *p* < 0.001. ** indicates *p* < 0.05), while there were statistically significant differences in the percentage of cells in two quadrants between C and A in U87MG cells by Student’s *t*-test (** indicates *p* < 0.05).

It was shown by Western blotting in LN229 and U87MG cells that either M_4_N or A_4_N suppressed the expression of SP1 but not MYC and HIF1A (however, it was previously shown that M_4_N alone could suppress the expression of HIF1A in LN229 cells under hypoxia conditions [10], and it was shown here that a small amount of the expression of HIF1A in U87MG cells was suppressed by A_4_N), while the combination treatment of M_4_N and A_4_N greatly suppressed the expression of all three of SP1, MYC, and HIF1A (Figure 2I). Meanwhile, it was shown that either M_4_N or A_4_N suppressed a small amount of the expression of some stem cell- and EM transition-related proteins, while the combination treatment of M_4_N and A_4_N greatly suppressed most of the stem cell- and EM transition-related proteins (Figure 2I). The data overall showed that (1) when the treatment (such as either M_4_N or A_4_N treatment alone) only partially suppressed the expression of SP1, MYC, or HIF1A, it suppressed only a small amount of the expression of both stem cell- and EM transition-related proteins and that (2) when the treatment (such as the combination treatment of M_4_N and A_4_N) almost totally suppressed the expression of SP1, MYC, and HIF1A, it greatly reduced the expression of both stem cell- and EM transition-related proteins at the same time.

The relationship between the three transcription factors (SP1, MYC, and HIF1A) and other proteins examined (stem cell- and EM transition-related proteins) was analyzed by the bioinformatic technique as well. The PPI network was constructed using the STRING database to visualize and analyze the functional relationships among these proteins. The resulting network demonstrated significant interconnectivity and clustering of the proteins of interest (Figure 2II). The PPI network consisted of 10 nodes (SP1, MYC, HIF1A, PROM1, BMI1, TERT, SOX2, SNAIL, TWIST, and ZEB1), representing individual proteins, connected by 44 edges, which indicate functional associations between the proteins. This network showed a high degree of interconnectedness, with an average node degree of eight, along with a high average local clustering coefficient of 0.885. This enrichment in interactions was further supported by a highly significant PPI enrichment *p*-value of <1.0 × 10^−16^, indicating that the proteins in our network have more interactions among themselves. These data (Figure 2I,II) overall supported our concept described above that the suppression of SP1, MYC, and HIF1A leads to the suppression of stem cell-related genes [15]. In addition, a GGI network was constructed with 10 nodes (*SP1*, *MYC*, *HIF1A*, *PROM1*, *BMI1*, *TERT*, *SOX2*, *SNAIL*, *TWIST*, and *ZEB1*) (Supplementary Figure S1). Functional enrichment analysis was conducted on the same ten genes to explore their associated functions (Supplementary Table S1).

### 3.3. Both M_4_N and A_4_N Alone Induced a Small Amount of Cell Death, While the Combination Treatment of M_4_N and A_4_N Induced a Large Amount of Cell Death in LN229 and U87MG Cells

Next, we studied the effect of M_4_N, A_4_N, or the combination treatment of M_4_N with A_4_N on the induction of cell death in LN229 and U87MG cells. Cell death was assayed by the fluorescence-activated cell sorting (FACS)-based analysis using Annexin V as an indicator for apoptosis and 7-AAD staining as an indicator for necrosis. The cell death analysis showed that M_4_N treatment alone did not change the number of either LN229 or U87MG cells in all quadrants (viable, apoptotic, necrotic, and necrotic+ apoptotic) that much, while A_4_N treatment alone increased the number of necrotic cells and decreased the number of viable cells in U87MG cells, although it did not change the number of LN229 cells in all quadrants that much (Figure 2III(a,b)). On the contrary, when either LN229 or U87MG cells were treated with M_4_N and A_4_N in combination, the treatment significantly increased the number of both necrotic and apoptotic cells, while it significantly decreased the number of viable cells (Figure 2III(a,b)). These data as well as the data shown in Figure 2I show that when the treatment reduced the expression of SP1, MYC, and HIF1A as well as that of stem cell- and EM transition-related proteins more, it induced more cell death. This supported our concept that the combination treatment of inhibitors for SP1, MYC, and HIF1A induced strong anticancer activity via its effect on suppressing the expression of stem cell- and EM transition-related proteins.

### 3.4. The Ontology Analysis Showed That the Proteins Associated with All Three Genes (SP1, MYC, and HIF1A) Were Likely Involved in Transcription Regulation for Both Coding and Non-Coding RNAs

So far, we have presented several pieces of evidence to support the concept that SP1, HIF1A, and MYC collaborate to promote cancer development and that inhibitors of these transcription factors should induce strong anticancer activity [15]. This led us to conduct a bioinformatics analysis on the proteins associated with SP1, MYC, and HIF1A.

**Figure 3:**
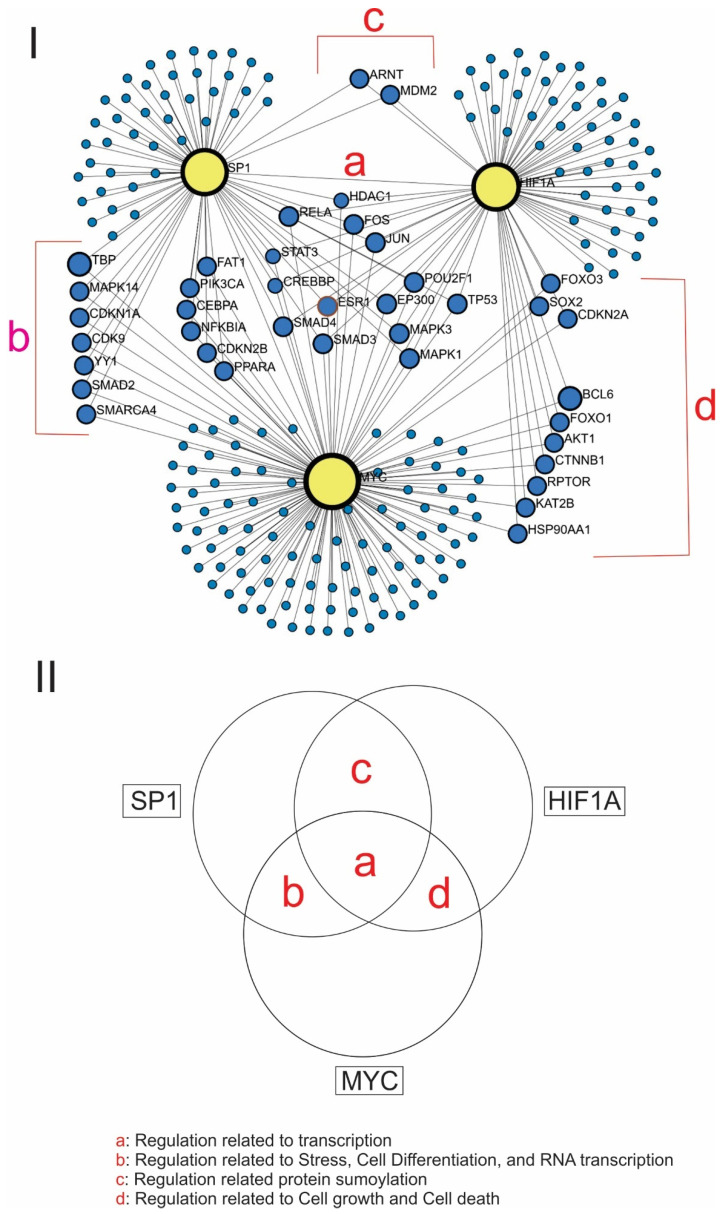
PPI network analysis of significant proteins associated with SP1, MYC, and HIF1A and the ontology analysis of these proteins. (**I**) Network Analysis: Construction of the PPI network of significant proteins for SP1, MYC, and HIF1A. (**II**) Ontology analysis of the proteins related to all three SP1, MYC, and HIF1A genes. The proteins related to SP1, MYC, and HIF1A are shown as group ‘a’. The proteins related to SP1 and MYC are shown as group ‘b’. The proteins related to SP1 and HIF1A are shown as group ‘c’. The proteins related to MYC and HIF1A are shown as group ‘d’. The ontology of each group is described inside the panel.

The PPI network analysis was performed for SP1, MYC, and HIF1A proteins (Figure 3). The resulting network comprised 230 nodes representing individual proteins and 283 edges representing the interactions between these proteins. The analysis (Figure 3I) showed that there were fourteen proteins that interacted with all three of SP1, MYC, and HIF1A, namely, MAPK1, CREBBP, MAPK3, EP300, STAT3, TP53, FOS, SMAD3, SMAD4, POU2F1, JUN, HDAC1, RELA, and ESR1. The ontogeny analysis of these proteins showed that all these proteins were involved in non-coding RNA-related regulation [33] and transcriptional regulation as shown (Group ‘a’ in Figure 3, Supplementary Figure S3A). Additionally, the network analysis showed that thirteen proteins (TBP, MAPK14, CDKN1A, CDK9, YY1, SMAD2, SMARCA4, FAT1, PIK3CA, CEBPA, NFKBIA, CDKN2B, and PPARA) interacted with both SP1 and MYC (Group ‘b’ in Figure 3, Supplementary Figure S3B). The ontogeny analysis showed that these proteins were involved in stress, cell differentiation, and the regulation of transcription. Further, two proteins (MDM2 and ARNT) interacted with both SP1 and HIF1A (Group ‘c’ in Figure 3, Supplementary Figure S3C). The ontology analysis showed that these proteins were involved in protein sumoylation. Finally, ten proteins (FOXO3, SOX2, CDKN2A, BCL6, FOXO1, AKT1, CTNNB1, RPTOR, KAT2B, and HSP90AA1) were revealed to interact with both MYC and HIF1A (Group ‘d’ in Figure 3, Supplementary Figure S3D). The ontology analysis of these protein was involved in cell cycle, growth, and cell death. In addition, the GGI network was constructed for *SP1*, *MYC,* and *HIF1A* through GeneMania as shown in Supplementary Figure S4. Pathway analysis was performed for SP1, MYC, and HIF1A (Supplementary Table S2).

### 3.5. A Gene-miRNA Network Analysis Revealed That There Were Seven miRNAs That Were Associated with all Three of SP1, MYC, and HIF1A

**Figure 4:**
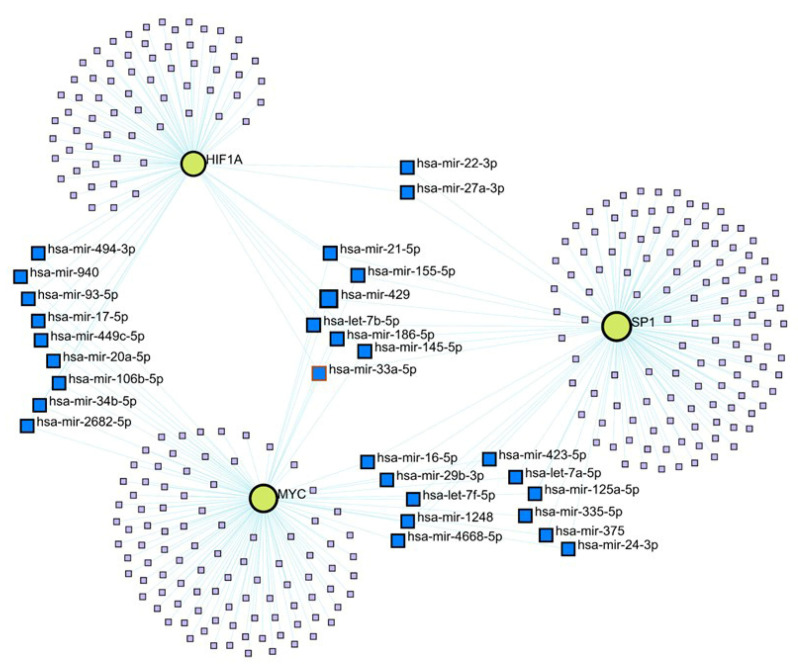
Construction of gene–miRNA interaction network of significant genes for SP1, MYC, and HIF1A. Gene–miRNA interaction networks were constructed for the genes SP1, MYC, and HIF1A by leveraging the miRTarBase v8.0 database. Seven miRNAs were shown to be associated with all three SP1, MYC, and HIF1A genes (hsa-mir-145/21/186/429/155/33a-5p and hsa-let-7b-5p). The identities of miRNAs that are associated with one of the three genes (SP1, MYC, and HIF1A) are listed in the supplement (Supplementary Table S3).

The PPI network analysis showed that the proteins associated with all three of SP1, MYC, and HIF1A are involved in the transcription regulation of both coding and non-coding RNAs (Figure 3). For the investigation of non-coding RNAs, we utilized miRNA-associated databases to focus specifically on the miRNAs [34] linked to SP1, MYC, and HIF1A. This analysis was conducted using the miRTarBase v8.0 database as a plugin within the NetworkAnalyst Tool (Figure 4). The resulting network of gene–miRNAs encompassed 3 hub genes (SP1, MYC, and HIF1A), 315 nodes, and 348 edges, representing the intricate interplay between these genes and their associated miRNAs. The analysis identified seven common miRNAs—specifically, hsa-miR-145, hsa-miR-21, hsa-miR-186, hsa-miR-429, hsa-miR-155, hsa-miR-33a-5p, and hsa-let-7b-5p—that are connected to all three transcription factors (SP1, MYC, and HIF1A).

## 4. Discussion

Previously, we proposed the concept that three major transcription factors, SP1, HIF1A, and MYC, collaborated together to promote the development of cancers, based on comprehensive investigations of various system analyses of cancer-related genes and promoter analyses of the *SP1*, *MYC*, and *HIF1A* genes, and that combination treatment with inhibitors of SP1, HIF1A, and MYC should suppress the expression of various genes associated with the regulation of cancer stem cells, which have key roles in the maintenance and progression of cancer cells, and should induce strong anticancer activity [15]. In this study, it was shown that the combination treatment of M_4_N with A_4_N markedly reduced the expression of the SP1, MYC, and HIF1A proteins as well as various cancer stem cell- and EM transition-related proteins in the LN229 and U87MG glioma cell lines and greatly induced cell death in both of these cell lines (Figure 2I,III). The PPI network analysis showed that there was a strong interconnectivity between three transcription factors (SP1, MYC, and HIF1A) and stem cell-related proteins (Figure 2II), which indicated that the suppression of the expression of SP1, MYC, and HIF1A most likely caused the reduction in the expression of these stem cell-related proteins. These data overall supported our hypothesis as mentioned above [15].

It was shown by multiple experiments that the promoter of the *SP1* gene contains numerous SP1 consensus sequences and that SP1 autoactivated the transcription of the *SP1* gene [15,35]. It has also been shown by multiple experiments that M_4_N suppressed the expression of various genes whose transcription levels were dependent on SP1 by competitively inhibiting the binding of SP1 transcription factors to the SP1 consensus sequences of the promoters of these genes [1,6,10,13,14,36]. Altogether, these data indicated that M_4_N reduced the transcription of the *SP1* gene by competitively blocking the binding of SP1 transcription factors to their consensus sequences in the *SP1* gene and then suppressed the expression of SP1 proteins in the majority of cancer cells including LN229 and U87MG cells (Figure 2I). On the other hand, the mechanism of the suppression of HIF1A and MYC protein expression by M_4_N is less obvious than that of SP1 protein, although there are numerous SP1 consensus sequences in the *HIF1A* gene promoter and some SP1 consensus sequences in the *MYC* gene promoter [15]. It was shown that SP1 induced HIF1A expression by upregulating the transcription of the *HIF1A* gene in several experiments [15], while M_4_N suppressed the expression of the HIF1A protein in LN229 and Hela cells [10]. However, the Northern blotting data showed that M_4_N did not modulate the level of HIF1A mRNA in Hela cells even though it greatly reduced the expression of HIF1A protein significantly [10]. These data indicated that M_4_N suppressed the expression of HIF1A non-transcriptionally and that the facilitation of the degradation of HIF1A protein was most likely involved in the mechanism by which M_4_N suppresses HIF1A protein expression [10]. On the contrary, unlike for the *SP1* or *HIF1A* gene, there is no direct evidence to suggest that SP1 directly affects the transcription of the *MYC* gene [15]. In fact, although the combination treatment of M_4_N and A_4_N suppressed the expression of MYC protein in LN229 and U87MG cells by unknown mechanisms, either M_4_N or A_4_N alone did not affect the expression in either LN229 or U87MG cells much (Figure 2I). In addition, it was shown that M_4_N suppressed the expression of *MYC* mRNA in LNCaP prostate cancer cells and AsPC1 pancreatic cancer cells but not in L428 leukemic cells when they were treated with M_4_N for several hours (Supplementary Table S4) [37,38]. Therefore, the data overall indicated that M_4_N was a dual inhibitor for SP1 and HIF1A and occasionally worked as an inhibitor for MYC in certain cancers.

The numerous data using tissue culture and mouse xenograft experiments have shown that the combination treatment of M_4_N with an appropriately selected second anticancer drug synergistically induced strong anticancer activity [25,28]. For instance, it was shown that M_4_N synergistically induced anticancer activity with either rapamycin or temozolomide [25,28]. Interestingly, either rapamycin or temozolomide has been shown to have activity to suppress MYC expression [39,40]. As discussed above, M_4_N is a dual inhibitor of SP1 and HIF1A but only occasionally works as a MYC inhibitor for certain cancer cells specifically. Thus, these experiments about combination drug therapy with M_4_N [25,28] suggested that M_4_N, a dual inhibitor of SP1 and HIF1A, induced anticancer activity effectively when it was combined with a drug that had activity to suppress MYC expression. This also supported our concept that the combination treatment of SP1, MYC, and HIF1A inhibitors should induce strong anticancer activity, and we previously suggested that the combination treatment of M_4_N with Omomyc, the only MYC inhibitor applicable to clinical trials, should effectively induce anticancer activity [15,41].

In this study, we also conducted bioinformatic analyses of the SP1, MYC, and HIF1A genes. It was shown by the ontology analysis of proteins that were selected by protein–protein analysis for SP1, MYC, and HIF1A that the proteins associated with all three of SP1, MYC, and HIF1A were involved in the regulation of transcription for either coding or non-coding RNAs (Figure 3). On the other hand, when the ontology analysis was performed on the proteins that were associated with either SP1/MYC, MYC/HIF1A, or HIF1A/SP1, it showed that these proteins were involved in only certain specific cellular metabolic mechanisms such as differentiation, cell growth, cell death, or others (Figure 3II). These data suggested that it might be possible to reduce the transcription and expression of the majority of the genes by inhibiting the expression of SP1, MYC, and HIF1A altogether. Since cancer cells require a great deal of production of both biological energy and building materials to renew cells for rapid proliferation, the expression of various genes needs to be enhanced far more in cancer cells than normal cells. Thus, the well-controlled combination treatment of SP1, MYC, and HIF1A inhibitors for cancer cells should keep the expression under control of the genes whose overexpression is necessary to satisfy the enormous appetite of cancer cells for their fast growth and renewal and should suppress cancer progression, whereas it should not induce a great negative impact on normal cells that do not require enormous amounts of energy and materials for their survival. Meanwhile, the PPI network analysis for the proteins related to stem cells and EM transition with SP1, MYC, and HIF1A showed that all these proteins had profound interactions with SP1, MYC, and HIF1A (Figure 2II). This suggested that the inhibition of SP1, MYC, and HIF1A should have strong negative impacts on the expression of stem cell- or EM transition-related cellular mechanisms that play important roles in the progression of cancer cells. Altogether, these bioinformatic data provided the theoretical background for our theory that the combination treatment of SP1, MYC, and HIF1A inhibitors should present strong anticancer efficacy.

As described, the PPI network analysis and the ontogeny analysis showed that the proteins associated with all three of SP1, MYC, and HIF1A were involved in the transcription for either coding or non-coding RNAs (Figure 3, Supplementary Figure S2). Furthermore, to examine the functional interactions between genes and miRNAs, we conducted a gene–miRNA network analysis (Figure 4), which produced an extensive network of 315 nodes. This indicated that each transcription factor did not only interact with its direct targets but also with a wide array of other genes through the action of miRNAs. This extensive connectivity underscores the potential for feedback loops and cross-regulatory mechanisms that can significantly influence cellular behavior in cancer. Given that SP1, MYC, and HIF1A are often overexpressed in tumors [15], the modulation of their expression by common miRNAs could be a critical factor in tumorigenesis.

In addition, the gene–miRNA analysis [42,43] (Figure 4) showed that there were seven miRNAs that were associated with all three of SP1, MYC, and HIF1A. The detailed analysis of these seven miRNAs based on various scientific literature reports supported our concept that the inhibitors of SP1, MYC, and HIF1A should result in the strong induction of anticancer activity (Table 1). Specifically, it was shown that mir-429 suppressed SP1, MYC, and HIF1A and that the induction of mir-429 was associated with apoptosis and the reduction of metastasis [44,45,46]. It was also shown that mir-145 suppressed all three of SP1, MYC, and HIF1A [47] as well and that the induction of mir-145 induced apoptosis [47]. Likewise, mir-186 suppressed SP1, MYC, and HIF1A, and the induction of mir-186 induces the sensitivity of cancer cells to anticancer drug [48,49,50]. In addition, microRNAs such as mir-155, mir-33a, and let-7b also suppress the expression of SP1, MYC, and HIF1A and induce anticancer activity [51,52,53,54,55,56,57,58,59]. On the contrary, mir-21 induces the expression of SP1, MYC, and HIF1A and promotes cancer progression via the induction of immune-suppressive mechanisms [60,61,62]. Thus, these miRNAs either positively or negatively affect cancer progression by targeting both cancer cells per se and the cancer microenvironment via modulation of all three of SP1, MYC, and HIF1A. Interestingly, it was shown that M_4_N had activity against the cancer microenvironment such as immune systems in addition to its direct activity against cancer cells, which suggested that there might be some connections between miRNAs and the activity of M_4_N in both cancers and the microenvironment [10,63]. Uniquely, mir-21 was the first miRNA that was recognized as a nuclear miRNA [64], which suggested that this miRNA could modulate not only the translation of some mRNAs but also the transcription of some genes. The existence of multiple miRNAs that specifically regulate all three of SP1, MYC, and HIF1A suggests that a set of transcription factors, SP1, MYC, and HIF1A, as a team has a key role in regulating some important cellular mechanisms. When some of these miRNAs suppress all of SP1, MYC, and HIF1A, they induce anticancer activity, while when others induce all three of SP1, MYC, and HIF1A, they induce procancer activity (Table 1). In addition, it was shown that there was an intricate interplay between cancer stem cells and miRNAs [65]. Our concept and the data presented here [15] (Figure 2, Figure 3 and Figure 4) indicated that SP1, MYC, and HIF1A were the missing links between miRNAs and cancer stem cells. These data overall strongly supported our concept and also indicated that the crucial mechanism for the intervention of cancer progression by the modulation of miRNAs was the suppression of all three of SP1, MYC, and HIF1A.

The characteristics of the miRNAs that were associated with all of SP1, MYC, and HIF1A according to the gene–miRNA network analysis (Figure 4) were examined by the scientific literature (the reference numbers are shown in the table). The column ‘Effect of miRNA on the expression of SP1/MYC/HIF1A’ indicates how each miRNA affects the expression of SP1, MYC, or HIF1A, as either ‘induction’ or ‘suppression’. The column ‘Activity of miRNA on cancers’ indicates how each miRNA affects cancers, as either ‘anticancer’ or ‘procancer’. All these data were obtained in various cancer cells and tumors, described in the references. As shown in the table, when a miRNA suppresses the expression of SP1, MYC, or HIF1A, this miRNA induces anticancer activity, whereas when a miRNA induces the expression of SP1, MYC, or HIF1A, this miRNA induces procancer activity. This suggests that these miRNAs affect cancers via their activity to either induce or suppress the expression of SP1, MYC, and HIF1A.

In addition to the experiments providing evidence to support our theory about the roles of SP1, MYC, and HIF1A in anticancer drug development [15], the efficacy of a new compound (A_4_N) was tested in this study (Figure 1B and Figure 2I,III). Although M_4_N showed strong anticancer efficacy when it was administered to the tumors directly, it showed only moderate anticancer activity when it was administered systemically [7,8,9]. This happened because it was hard to dissolve M_4_N in aqueous solutions including serum due to its lipophilic characteristics so that it was not easy to increase the M_4_N concentration in the serum after administration of the drug either intravenously or orally. Unlike M_4_N, A_4_N can be dissolved in water without issues due to its more hydrophilic characteristics than M_4_N thanks to its four acetylate groups (Figure 1B). The cell death data overall showed that A_4_N had a little bit stronger cytotoxic activity than M_4_N and that the combination treatment of M_4_N with A_4_N induced a great deal of cell death (Figure 2III). Since M_4_N can accumulate in organs due to its lipophilic nature by continuous feeding of the drug [10], it has its own advantage over A_4_N. Meanwhile, the advantage of A_4_N is its good solubility in water. The data overall suggested that the combination treatment of M_4_N and A_4_N could represent a better anticancer regimen than M_4_N or A_4_N alone (Figure 2III).

## Figures and Tables

**Table 1:** The correlation between the effect of miRNAs on the expression of SP1, MYC, and HIF1A and the activity of miRNAs on cancer.

miRNA	Effect of miRNA on the Expression of SP1/MYC/HIF1A	Activity of miRNA on Cancers	Reference
mir-429	Suppression	Anticancer	[44,45,46]
mir-145	Suppression	Anticancer	[47]
mir-186	Suppression	Anticancer	[48,49,50]
mir-155	Suppression	Anticancer	[51,52,53]
mir-33a	Suppression	Anticancer	[54,55,56]
let-7b	Suppression	Anticancer	[57,58,59]
mir-21	Induction	Procancer	[60,61,62]

## Data Availability

Data is available upon request.

## Supplementary Materials

The following supporting information can be downloaded at: https://www.mdpi.com/article/10.3390/cimb46110741/s1, Figure S1: Construction of GGI Network through GeneMania Database; Table S1. Functional Enrichment Analysis; Figure S2. The full images of the blots used for the western blotting data in Figure 2I; Figure S3. The ontology analysis of the proteins associated with all SP1, MYC, and HIF1A (group A), SP1 and MYC (group B), SP1 and HIF1A (group C), and MYC and HIF1A (group D); Figure S4. Construction of GGI network of significant genes for SP1, MYC and HIF1A; Table S2. List of most significantly enriched pathways of SP1, MYC, and HIF1A, analyzed by Reactome Pathway analysis; Table S3. The miRNAs associated with one, two, or all of three transcription factors SP1, MYC, and HIF1A; Table S4. Effect of M4N on the expression of MYC mRNA in LNCaP prostate, AsPC1 pancreatic, and L428 leukemic cells at 6hrs after treatment of M_4_N.
