# Supplementary material for: Evidence to Support the Collaboration of SP1, MYC, and HIF1A and Their Association with microRNAs
Source: Curr Issues Mol Biol. 2024 Nov 5;46(11):12481–96. doi: 10.3390/cimb46110741 (PMC11592871; doi:10.3390/cimb46110741)
Supplement: Supplementary file 1 [file cimb-46-00741-s001.zip › cimb-3275201-supplementary.pdf]

SUPPLEMENT:

Supplemental Figure S1. Construction of GGI Network through GeneMania Database.

To further elucidate the functional relationships and interactions among the ten genes coding the proteins which were detected by the western blotting **(Figure 2I)**, we employed the GeneMANIA tool to construct a comprehensive gene interaction network. A GGI network was constructed with 10 nodes. Circular black coloured nodes with lines represent significant genes (*SP1, MYC, HIF1A, PROM1, BMI1, TERT, SOX2, SNAIL, TWIST, ZEB1*) **(Supplementary Figure S1).** Coloured edges represent the interaction between the genes, including, physical interaction - pink; genetic interaction - green; predicted - orange; shared protein domain - grey; pathway - light blue; co-localization - dark blue. The size of the nodes and the width of the edges reflect the strength of the interaction.

This analysis revealed several hub genes that demonstrated significant connectivity through various types of interactions, including co-expression, predicted functional associations, physical interactions, and genetic interactions. In addition, Functional enrichment analysis is performed **(Supplementary Table S1)**. In this analysis we performed pathway analysis **(Supplementary Table S1A).** The analysis revealed important enriched pathways, including signaling by ALK, SMAD2/SMAD3: SMAD4 heterotrimer regulates transcription, Transcriptional regulation of pluripotent stem cells, and Interleukin-4 and Interleukins-13 signaling. Followed by Disease Ontology analysis, the enriched DO terms indicated that the identified genes were associated with several diseases, including cancer, nasopharyngeal disease, GBM multiforme, tumors of the exocrine pancreas, and colorectal cancer **(Supplementary Table S1B)**. GO enrichment analysis was performed to elucidate the functional characteristics of the

identified genes. The results were stratified into three primary GO categories: biological processes (BP), molecular functions (MF), and cellular components (CC) **(Supplementary Table S1C)**.

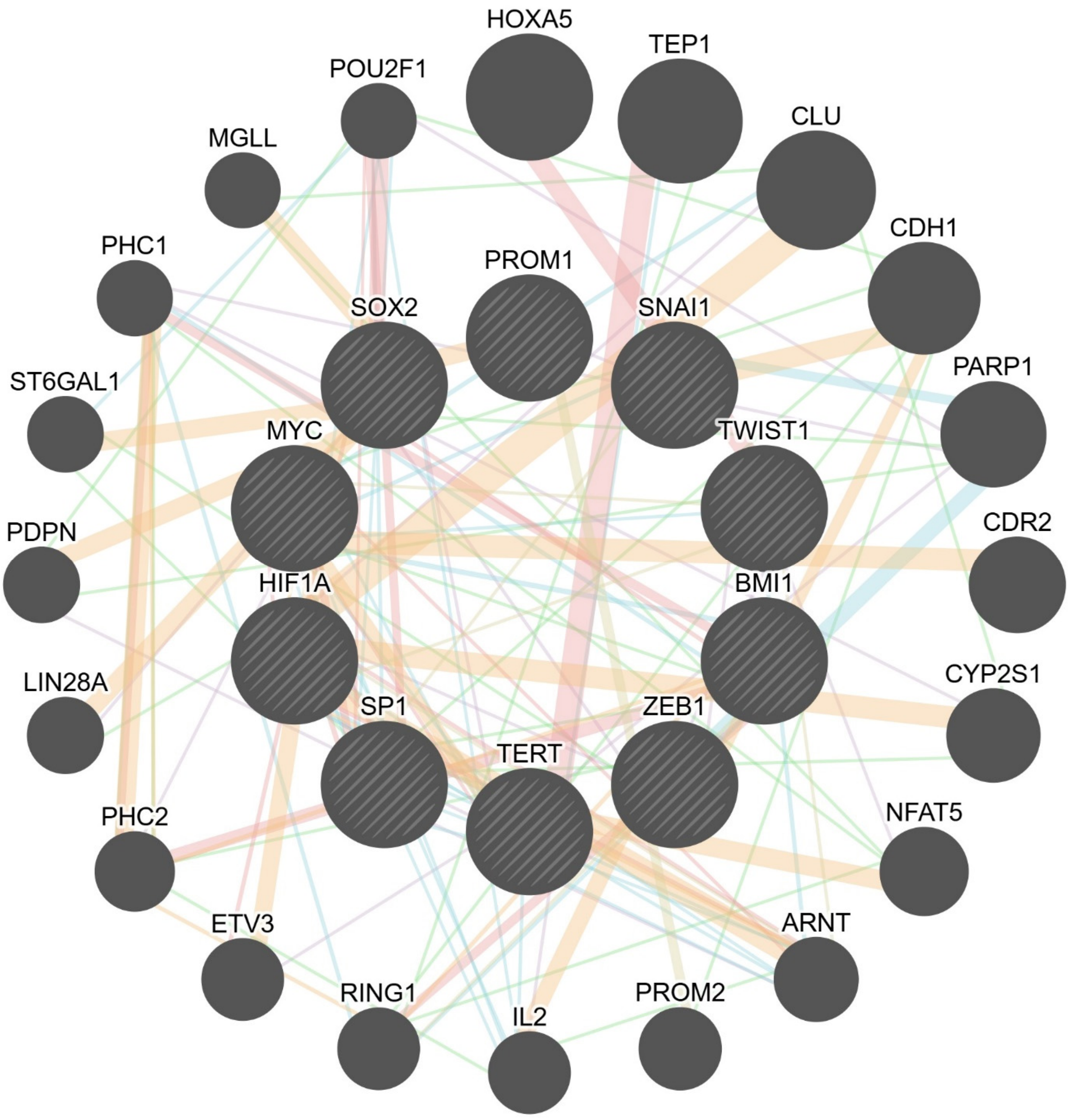

Supplemental Table S1. Functional Enrichment Analysis:

Table S1A: Pathway Analysis:

S.No.	Gene Set	Description	Hit genes	p-Value	FDR
1	R-HSA-6785807	Interleukin-4 and Interleukin-13 signaling	MYC, HIF1A, ZEB1, TWIST1, SOX2	0.00	0.00
2	R-HSA-449147	Signaling by Interleukins	MYC, HIF1A, ZEB1, TWIST1, SOX2	0.00	0.01
3	R-HSA-9762293	Regulation of CDH11 gene transcription	SP1, SNAI1	0.00	0.01
4	R-HSA-1280215	Cytokine Signaling in Immune system	MYC, HIF1A, ZEB1, TWIST1, SOX2	0.00	0.04
5	R-HSA-201556	Signaling by ALK	MYC, HIF1A	0.00	0.05
6	R-HSA-9759475	Regulation of CDH11 Expression and Function	SP1, SNAI1	0.00	0.05
7	R-HSA-9759476	Regulation of Homotypic Cell-Cell Adhesion	SP1, SNAI1	0.00	0.05
8	R-HSA-9764260	Regulation of Expression and Function of Type II Classical Cadherins	SP1, SNAI1	0.00	0.05
9	R-HSA-2173796	SMAD2/SMAD3:SMAD4 heterotrimer regulates transcription	SP1, MYC	0.00	0.06
10	R-HSA-2122947	NOTCH1 Intracellular Domain Regulates Transcription	MYC, HIF1A	0.00	0.10

Table S1B: Disease Ontology:

S.No.	Gene Set	Description	Hit genes	p-Value	FDR
1	umls:C0027626	Neoplasm Invasiveness	SP1, HIF1A, ZEB1, TWIST1, SNAI1	0.00	0.00
2	umls:C0017636	Glioblastoma	MYC, HIF1A, PROM1, BMI1	0.00	0.00
3	umls:C0007621	Neoplastic Cell Transformation	SP1, MYC, ZEB1, SNAI1	0.00	0.00
4	umls:C0027627	Neoplasm Metastasis	SP1, MYC, TWIST1, SNAI1	0.00	0.00
5	umls:C0029463	Osteosarcoma	MYC, BMI1	0.00	0.12
6	umls:C0037286	Skin Neoplasms	SP1, HIF1A	0.00	0.20
7	umls:C0038356	Stomach Neoplasms	MYC, TWIST1, SNAI1	0.00	0.34
8	umls:C0030297	Pancreatic Neoplasm	MYC, HIF1A	0.00	0.34
9	umls:C0279626	Squamous cell carcinoma of esophagus	HIF1A, SOX2	0.00	0.34
10	umls:C0919267	ovarian neoplasm	MYC, ZEB1	0.01	0.34

Table S1C: Gene Ontology:

The GO enrichment analysis was performed to elucidate the functional characteristics of the identified genes. The results were stratified into three primary GO categories: biological processes (BP), molecular functions (MF), and cellular components (CC) **(Supplementary Table S1C)**. In the BP category, significant enrichment was observed in several key processes, including positive regulation of developmental processes, positive regulation of miRNA transcription,

negative regulation of RNA metabolic processes, and regulation of cell differentiation **(Supplementary Table S1Ca)**. These findings suggest a potential role for the identified genes in modulating developmental pathways and gene expression regulation. Analysis of the MF category revealed functional enrichment in transcription factor binding, sequence-specific DNA binding, and E-box binding **(Supplementary Table S1Cb)**. This enrichment pattern indicates that the identified genes may play crucial roles in transcriptional regulation and DNA-protein interactions. Within the CC category, the genes exhibited significant enrichment in chromatin, nucleoplasm, and chromosome-associated components **(Supplementary Table S1Cc)**. This localization pattern suggests that the identified genes may be involved in nuclear processes and chromatin-mediated regulation.

S1Ca: Biological Process GO:

S.No.	Gene Set	Description	Hit genes	p-Value	FDR
1	GO:0051094	positive regulation of developmental process	SP1, MYC, HIF1A, ZEB1, TWIST1, PROM1, SOX2, SNAI1	0.00	0.00
2	GO:0050678	regulation of epithelial cell proliferation	SP1, MYC, HIF1A, ZEB1, TWIST1, SOX2	0.00	0.00
3	GO:0050673	epithelial cell proliferation	SP1, MYC, HIF1A, ZEB1, TWIST1, SOX2	0.00	0.00
4	GO:0048593	camera-type eye morphogenesis	HIF1A, ZEB1, TWIST1, PROM1	0.00	0.00
5	GO:0001654	eye development	HIF1A, ZEB1, TWIST1, PROM1, SOX2	0.00	0.00

6	GO:0150063	visual system development	HIF1A, ZEB1, TWIST1, PROM1, SOX2	0.00	0.00
7	GO:0048880	sensory system development	HIF1A, ZEB1, TWIST1, PROM1, SOX2	0.00	0.00
8	GO:0045595	regulation of cell differentiation	MYC, HIF1A, ZEB1, TWIST1, PROM1, SOX2, SNAI1	0.00	0.00
9	GO:0045597	positive regulation of cell differentiation	HIF1A, ZEB1, TWIST1, PROM1, SOX2, SNAI1	0.00	0.00
10	GO:0048592	eye morphogenesis	HIF1A, ZEB1, TWIST1, PROM1	0.00	0.00

S1Cb: Molecular Function GO:

S.No.	Gene Set	Description	Hit genes	p-Value	FDR
1	GO:0070888	E-box binding	MYC, HIF1A, ZEB1, TWIST1, SNAI1	0.00	0.00
2	GO:0000978	RNA polymerase II cis-regulatory region sequence-specific DNA binding	SP1, MYC, HIF1A, ZEB1, TWIST1, SOX2, SNAI1	0.00	0.00
3	GO:0000987	cis-regulatory region sequence-specific DNA binding	SP1, MYC, HIF1A, ZEB1, TWIST1, SOX2, SNAI1	0.00	0.00
4	GO:0000981	DNA-binding transcription factor activity, RNA polymerase II-specific	SP1, MYC, HIF1A, ZEB1, TWIST1, SOX2, SNAI1	0.00	0.00
5	GO:0001221	transcription coregulator binding	SP1, MYC, HIF1A, TWIST1	0.00	0.00

6	GO:0001217	DNA-binding transcription repressor activity	MYC, HIF1A, ZEB1, TWIST1, SNAI1	0.00	0.00
7	GO:0000977	RNA polymerase II transcription regulatory region sequence-specific DNA binding	SP1, MYC, HIF1A, ZEB1, TWIST1, SOX2, SNAI1	0.00	0.00
8	GO:0003700	DNA-binding transcription factor activity	SP1, MYC, HIF1A, ZEB1, TWIST1, SOX2, SNAI1	0.00	0.00
9	GO:0000976	transcription cis-regulatory region binding	SP1, MYC, HIF1A, ZEB1, TWIST1, SOX2, SNAI1	0.00	0.00
10	GO:0001067	transcription regulatory region nucleic acid binding	SP1, MYC, HIF1A, ZEB1, TWIST1, SOX2, SNAI1	0.00	0.00

S1Cc: Cellular Component GO:

S.No.	Gene Set	Description	Hit genes	p-Value	FDR
1	GO:0000785	chromatin	SP1, MYC, HIF1A, ZEB1, TWIST1, SOX2, SNAI1	0.00	0.00
2	GO:0032993	protein-DNA complex	SP1, MYC, HIF1A, ZEB1, TWIST1, SOX2, SNAI1	0.00	0.00
3	GO:0005694	chromosome	SP1, MYC, HIF1A, ZEB1, TWIST1, SOX2, SNAI1	0.00	0.00
4	GO:0005667	transcription regulator complex	SP1, MYC, HIF1A, SOX2	0.00	0.01

5	GO:0000791	euchromatin	SP1, HIF1A	0.00	0.07
6	GO:0017053	transcription repressor complex	SP1, MYC	0.00	0.10
7	GO:0090575	RNA polymerase II transcription regulator complex	MYC, HIF1A	0.00	0.80
8	GO:0090571	RNA polymerase II transcription repressor complex	MYC	0.01	0.92
9	GO:0042622	photoreceptor outer segment membrane	PROM1	0.01	0.92
10	GO:0005721	pericentric heterochromatin	SNAI1	0.01	1.00

Supplemental Figure S2. The full images of the blots used for the western blotting data in Figure 2I. LN229 and U87MG cells were treated with M_4N (60μM) and/or A_4N (60μM) for 48hrs and the contents of each protein was examined by the western blotting. C: Control, M: M_4N (60μM), A: A_4N (60μM), and MA: M_4N (60μM) and A_4N (60μM) combination treatment. The arrows indicate the bands corresponding to the target proteins.

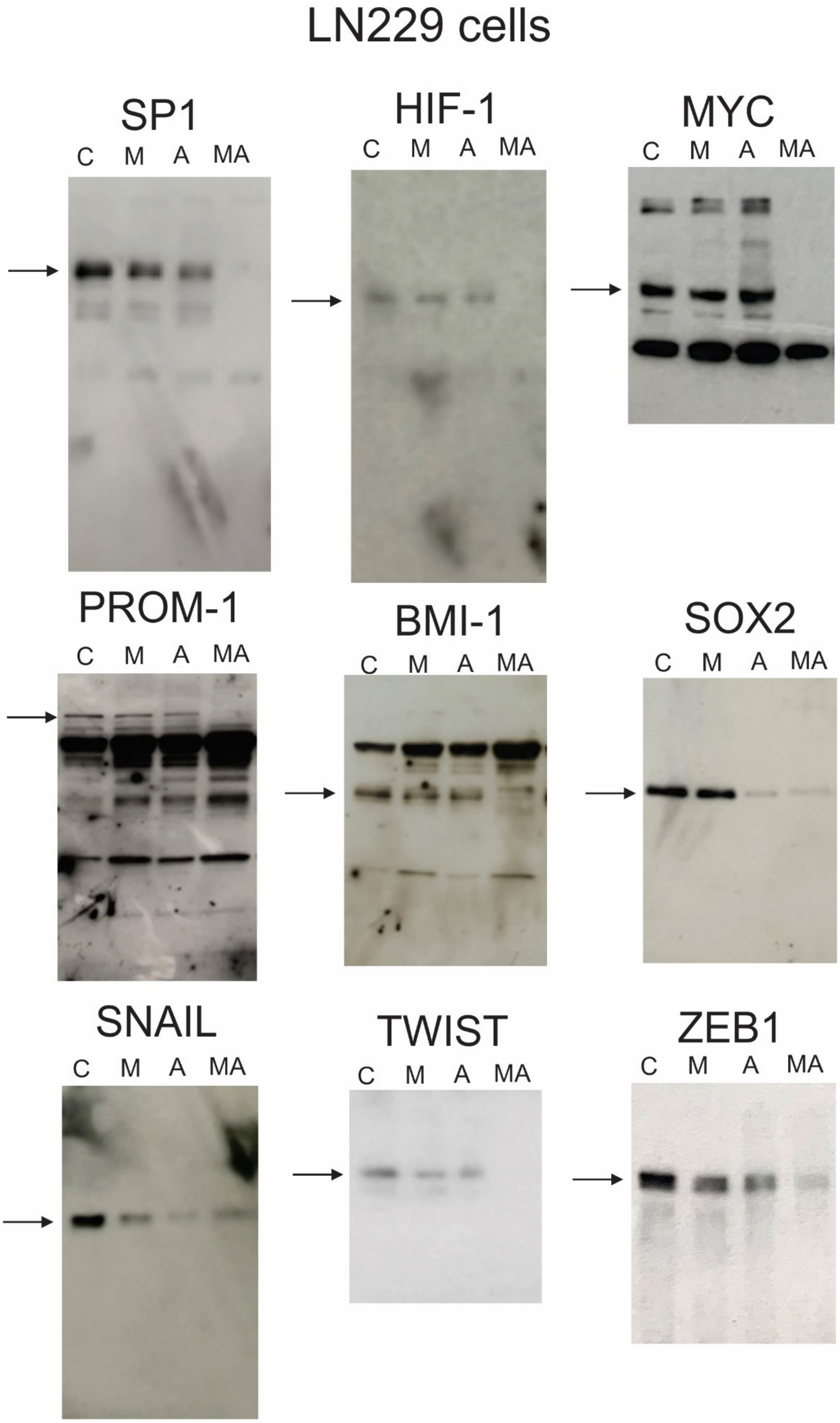

LN229 cells
SP1
C M A MA
HIF-1
C M A MA
MYC
C M A MA
PROM-1
C M A MA
BMI-1
C M A MA
SOX2
C M A MA
SNAIL
C M A MA
TWIST
C M A MA
ZEB1
C M A MA

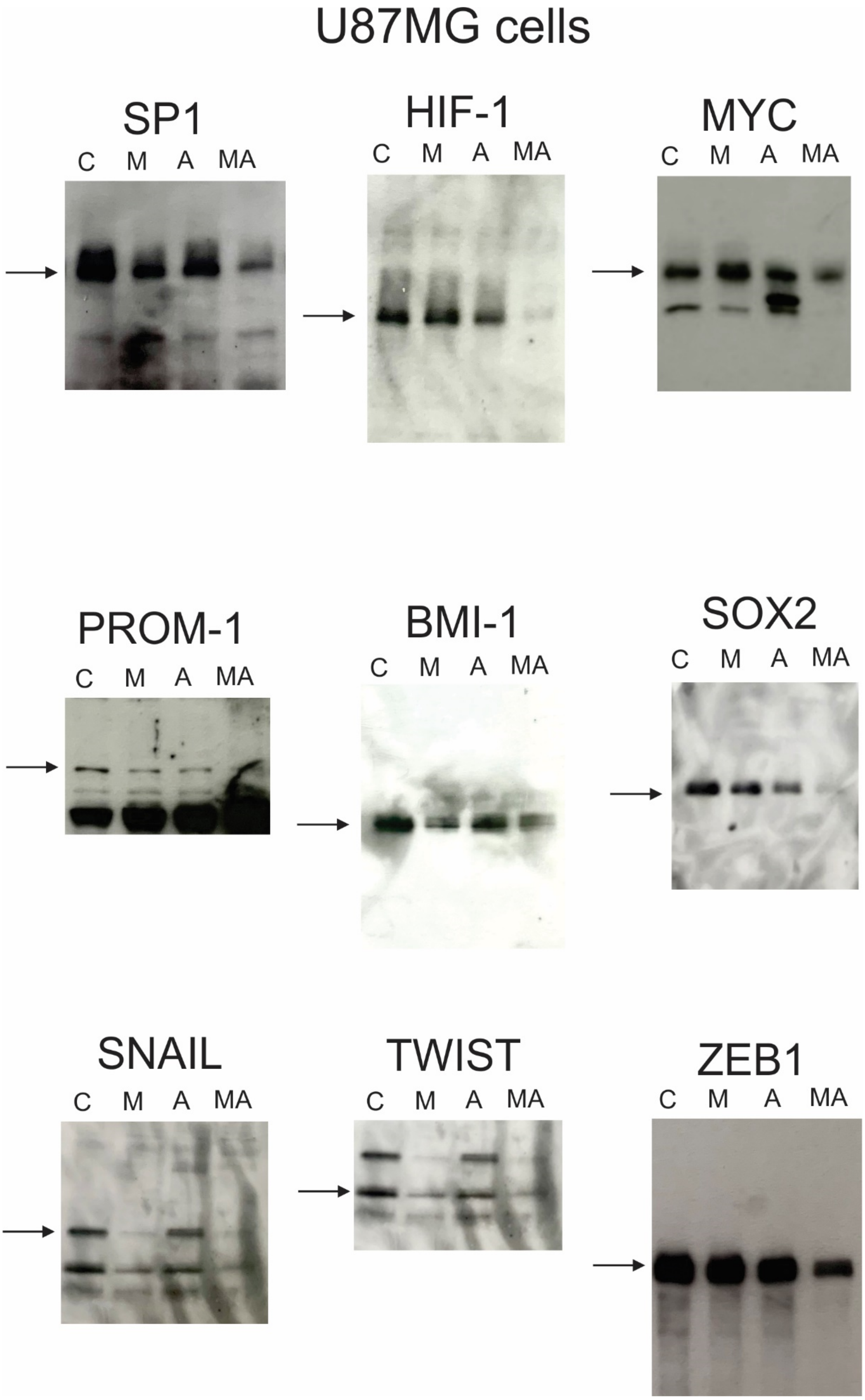
U87MG cells
SP1
C M A MA
HIF-1
C M A MA
MYC
C M A MA
PROM-1
C M A MA
BMI-1
C M A MA
SOX2
C M A MA
SNAIL
C M A MA
TWIST
C M A MA
ZEB1
C M A MA

Supplemental Figure S3. The ontology analysis of the proteins associated with all SP1, MYC, and HIF1A (group A), SP1 and MYC (group B), SP1 and HIF1A (group C), and MYC and HIF1A (group D).

Supplemental Figure S3A - Group A: Common genes associated with SP1, MYC & HIF1A (MAPK1, CREBBP, MAPK3, EP300, STAT3, TP53, FOS, SMAD3, SMAD4, POU2F1, JUN, HDAC1, RELA, ESR1)

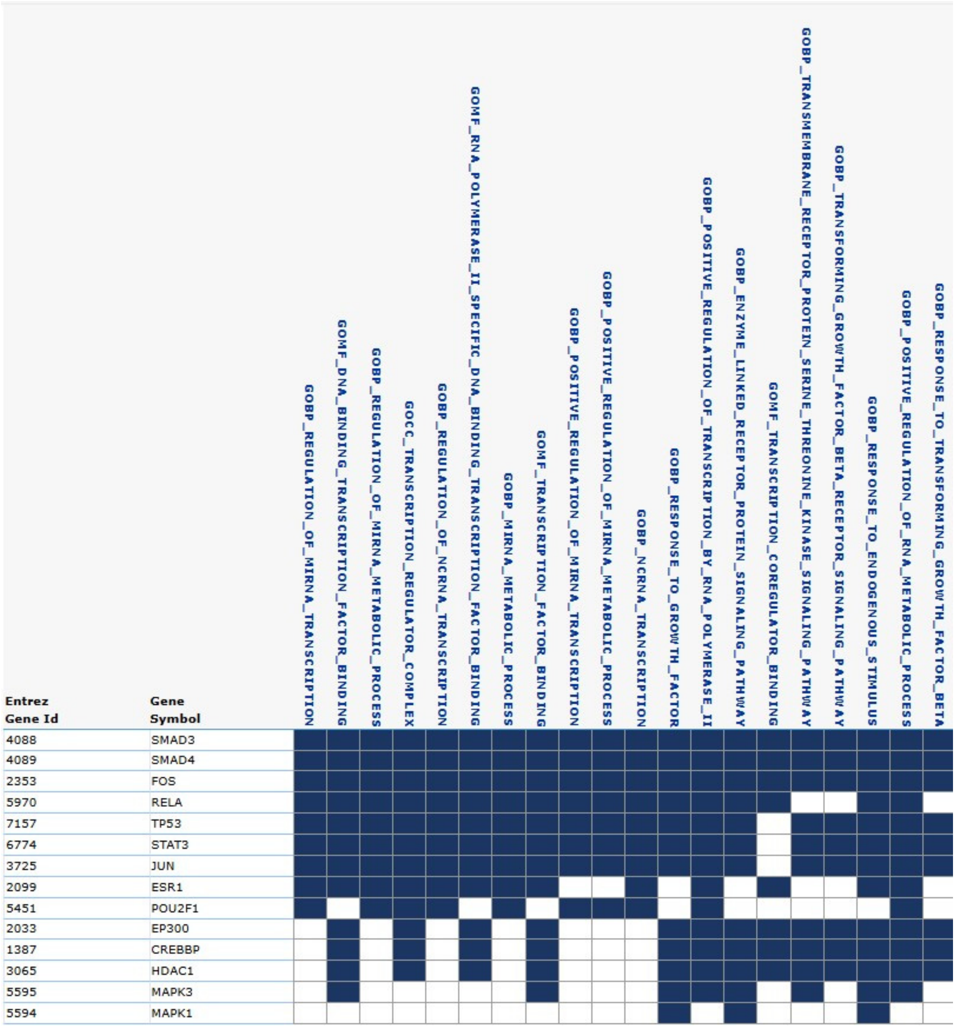

Supplemental Figure S3B - Group B: Common genes associated with SP1 & MYC

(TBP, MAPK14, CDKN1A, CDK9, YY1, SMAD2, SMARCA4, FAT1, PIK3CA, CEBPA, NFKBIA, CDKN2B, PPARA)

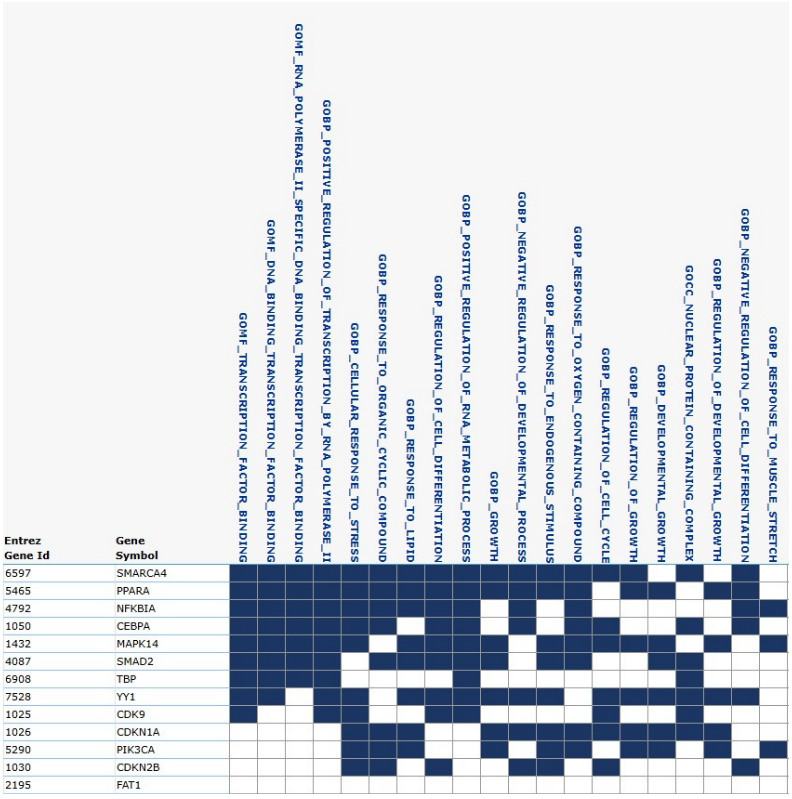

Supplemental Figure S3C - Group C: Common genes associated with SP1 & HIF1A

(MDM2, ARNT)

Entrez Gene Id	Gene Symbol	GOBP_PROTEIN_SUMOYLATION
4193	MDM2	■
405	ARNT	■

Supplemental Figure S3D - Group D: Common genes associated with MYC & HIF1A

(FOXO3, SOX2, CDKN2A, BCL6, FOXO1, AKT1, CTNNB1, RPTOR, KAT2B, HSP90AA1)

Entrez Gene Id	Gene Symbol	GOBP_REGULATION_OF_CELL_CYCLE_PROCESS	GOMF_KINASE_BINDING	GOMF_PROTEIN_SERINE_THREONINE_KINASE_INHIBITOR_ACTIVITY	GOBP_REGULATION_OF_PROGRAMMED_CELL_DEATH	GOBP_GROWTH	GOBP_CELL_GROWTH	GOBP_RESPONSE_TO_EXTRACELLULAR_STIMULUS	GOBP_APOPTOTIC_PROCESS	GOBP_CELLULAR_RESPONSE_TO_STRESS	GOBP_REGULATION_OF_CELL_CYCLE	GOBP_POSITIVE_REGULATION_OF_CATABOLIC_PROCESS	GOMF_KINASE_INHIBITOR_ACTIVITY	GOBP_POSITIVE_REGULATION_OF_CARBOHYDRATE_METABOLIC_PROCESS	GOBP_CELL_CYCLE_PROCESS	GOBP_NEGATIVE_REGULATION_OF_RNA_BIOSYNTHETIC_PROCESS	GOBP_REGULATION_OF_TRANSFERASE_ACTIVITY	GOBP_CELLULAR_RESPONSE_TO_CHEMICAL_STRESS	GOBP_REGULATION_OF_CATALYTIC_ACTIVITY	GOBP_NEGATIVE_REGULATION_OF_NUCLEOBASE_CONTAINING_COMPOUND_METABOLIC_PROCESS	GOBP_REGULATION_OF_CELL_DIFFERENTIATION
207	AKT1	■	■	■	■	■	■	■	■	■	■	■	■	■	■		■	■	■		■
1029	CDKN2A	■	■	■	■	■	■		■	■	■		■		■	■	■		■	■	
57521	RPTOR	■	■	■		■	■	■		■	■	■	■	■	■		■	■	■		■
8850	KAT2B	■	■	■				■		■	■	■	■	■	■	■	■	■	■	■	
1499	CTNNB1	■	■		■	■	■		■		■				■	■	■		■	■	■
604	BCL6	■			■	■	■		■	■	■				■	■				■	■
6657	SOX2	■			■	■		■	■		■				■	■			■	■	■
3320	HSP90AA1		■		■	■	■		■	■		■					■		■		
2309	FOXO3		■		■			■	■	■		■				■		■		■	■
2308	FOXO1				■			■	■	■		■		■		■		■		■	■

Supplemental Figure S4. Construction of GGI network of significant genes for SP1, MYC and HIF1A.

Circular black coloured nodes with lines represent significant genes (*MYC, SP1 & HIF1A*) and the circular nodes represent interacting genes. Coloured edges represent the interaction between the genes, physical interaction - pink; genetic interaction - green; predicted - orange; co-expression - purple; shared protein domain - grey; pathway - light blue; co-localization - dark blue. The size of the nodes and the width of the edges reflect the strength of the interaction.

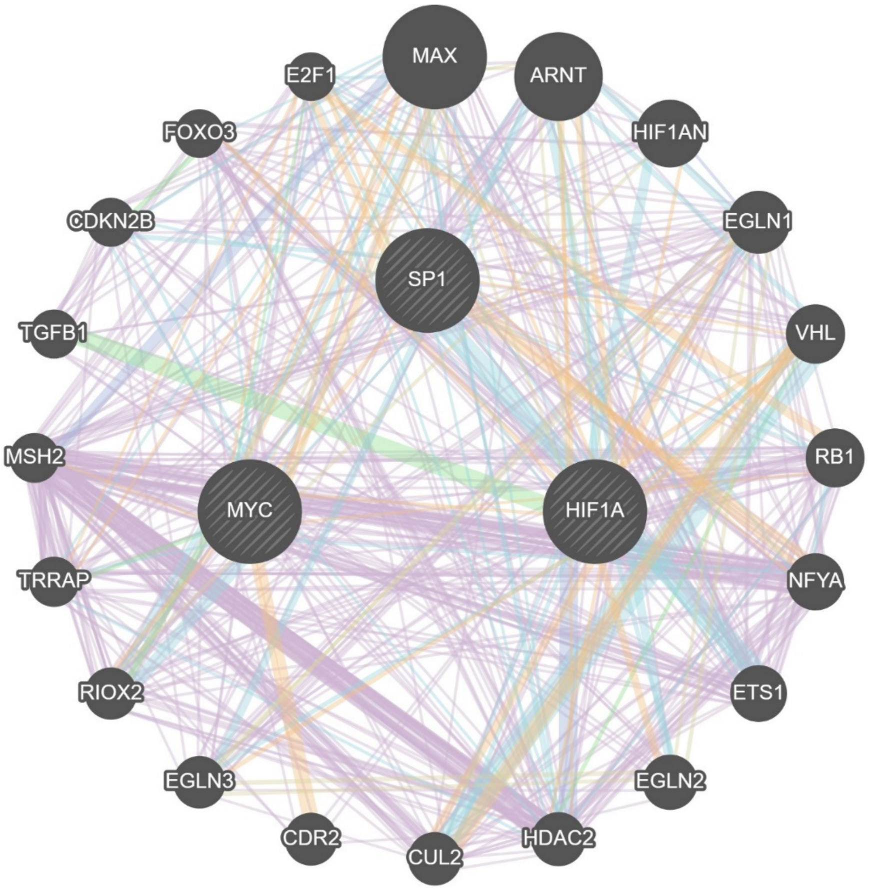

Supplemental Table S2. List of most significantly enriched pathways of SP1, MYC, and HIF1A, analyzed by Reactome Pathway analysis.

***FDR: False Discovery Rate**

S.No.	Gene Set	Description	P Value	FDR	Hit genes
1	R-HSA-201556	Signaling by ALK	0.00	0.03	HIF1A, MYC
2	R-HSA-2173796	SMAD2/SMAD3: SMAD4 heterotrimer regulates transcription	0.00	0.03	MYC, SP1
3	R-HSA-2122947	NOTCH1 Intracellular Domain Regulates Transcription	0.00	0.03	HIF1A, MYC
4	R-HSA-2173793	Transcriptional activity of SMAD2/SMAD3: SMAD4 heterotrimer	0.00	0.03	MYC, SP1
5	R-HSA-1980143	Signaling by NOTCH1	0.00	0.05	HIF1A, MYC
6	R-HSA-170834	Signaling by TGF-beta Receptor Complex	0.00	0.07	MYC, SP1
7	R-HSA-9759194	Nuclear events mediated by NFE2L2	0.00	0.07	MYC, SP1
8	R-HSA-6785807	Interleukin-4 and Interleukin-13 signaling	0.00	0.07	HIF1A, MYC
9	R-HSA-9006936	Signaling by TGFB family members	0.00	0.07	MYC, SP1

10	R-HSA-2262752	Cellular responses to stress	0.00	0.07	MYC, SP1, HIF1A

Supplemental Table S3. The miRNAs associated with one, two, or all of three transcription factors SP1, MYC, and HIF1A. The associations indicate which one, two, or all of three transcription factors are associated to each specific miRNA in miRNA database (miRTarBase v8.0).

Id	Label	Degree	Associations	Betweenness
6667	**SP1**	142		31274.47
4609	**MYC**	122		26701.18
3091	**HIF1A**	84		19054.35
MIMAT0000063	hsa-let-7b-5p	3	SP1 MYC HIF1A	2125.92
MIMAT0000076	hsa-mir-21-5p	3	SP1 MYC HIF1A	2125.92
MIMAT0000091	hsa-mir-33a-5p	3	SP1 MYC HIF1A	2125.92
MIMAT0000437	hsa-mir-145-5p	3	SP1 MYC HIF1A	2125.92
MIMAT0000456	hsa-mir-186-5p	3	SP1 MYC HIF1A	2125.92
MIMAT0000646	hsa-mir-155-5p	3	SP1 MYC HIF1A	2125.92
MIMAT0001536	hsa-mir-429	3	SP1 MYC HIF1A	2125.92
MIMAT0000077	hsa-mir-22-3p	2	MYC HIF1A	986.15
MIMAT0000084	hsa-mir-27a-3p	2	MYC HIF1A	986.15
MIMAT0000062	hsa-let-7a-5p	2	SP1 MYC	702.65
MIMAT0000067	hsa-let-7f-5p	2	SP1 MYC	702.65
MIMAT0000069	hsa-mir-16-5p	2	SP1 MYC	702.65
MIMAT0000080	hsa-mir-24-3p	2	SP1 MYC	702.65
MIMAT0000100	hsa-mir-29b-3p	2	SP1 MYC	702.65
MIMAT0000443	hsa-mir-125a-5p	2	SP1 MYC	702.65
MIMAT0000728	hsa-mir-375	2	SP1 MYC	702.65
MIMAT0000765	hsa-mir-335-5p	2	SP1 MYC	702.65
MIMAT0004748	hsa-mir-423-5p	2	SP1 MYC	702.65
MIMAT0005900	hsa-mir-1248	2	SP1 MYC	702.65
MIMAT0019745	hsa-mir-4668-5p	2	SP1 MYC	702.65
MIMAT0000070	hsa-mir-17-5p	2	HIF1A SP1	437.13
MIMAT0000075	hsa-mir-20a-5p	2	HIF1A SP1	437.13
MIMAT0000093	hsa-mir-93-5p	2	HIF1A SP1	437.13
MIMAT0000680	hsa-mir-106b-5p	2	HIF1A SP1	437.13
MIMAT0000685	hsa-mir-34b-5p	2	HIF1A SP1	437.13
MIMAT0002816	hsa-mir-494-3p	2	HIF1A SP1	437.13
MIMAT0004983	hsa-mir-940	2	HIF1A SP1	437.13
MIMAT0010251	hsa-mir-449c-5p	2	HIF1A SP1	437.13
MIMAT0013517	hsa-mir-2682-5p	2	HIF1A SP1	437.13
MIMAT0000242	hsa-mir-129-5p	1	SP1	0
MIMAT0000265	hsa-mir-204-5p	1	SP1	0
MIMAT0000268	hsa-mir-211-5p	1	SP1	0
MIMAT0000275	hsa-mir-218-5p	1	SP1	0
MIMAT0000280	hsa-mir-223-3p	1	SP1	0
MIMAT0000318	hsa-mir-200b-3p	1	SP1	0
MIMAT0000416	hsa-mir-1-3p	1	SP1	0
MIMAT0000419	hsa-mir-27b-3p	1	SP1	0

MIMAT0000422	hsa-mir-124-3p	1	SP1	0
MIMAT0000424	hsa-mir-128-3p	1	SP1	0
MIMAT0000427	hsa-mir-133a-3p	1	SP1	0
MIMAT0000429	hsa-mir-137	1	SP1	0
MIMAT0000450	hsa-mir-149-5p	1	SP1	0
MIMAT0000451	hsa-mir-150-5p	1	SP1	0
MIMAT0000681	hsa-mir-29c-3p	1	SP1	0
MIMAT0000689	hsa-mir-99b-5p	1	SP1	0
MIMAT0000690	hsa-mir-296-5p	1	SP1	0
MIMAT0000721	hsa-mir-369-3p	1	SP1	0
MIMAT0000727	hsa-mir-374a-5p	1	SP1	0
MIMAT0000731	hsa-mir-378a-5p	1	SP1	0
MIMAT0000751	hsa-mir-330-3p	1	SP1	0
MIMAT0000756	hsa-mir-326	1	SP1	0
MIMAT0000761	hsa-mir-324-5p	1	SP1	0
MIMAT0000770	hsa-mir-133b	1	SP1	0
MIMAT0001629	hsa-mir-329-3p	1	SP1	0
MIMAT0002171	hsa-mir-410-3p	1	SP1	0
MIMAT0002811	hsa-mir-202-3p	1	SP1	0
MIMAT0002862	hsa-mir-527	1	SP1	0
MIMAT0003271	hsa-mir-603	1	SP1	0
MIMAT0003280	hsa-mir-612	1	SP1	0
MIMAT0003294	hsa-mir-625-5p	1	SP1	0
MIMAT0004598	hsa-mir-141-5p	1	SP1	0
MIMAT0004612	hsa-mir-186-3p	1	SP1	0
MIMAT0004683	hsa-mir-362-3p	1	SP1	0
MIMAT0004918	hsa-mir-892b	1	SP1	0
MIMAT0004955	hsa-mir-374b-5p	1	SP1	0
MIMAT0004972	hsa-mir-922	1	SP1	0
MIMAT0005457	hsa-mir-518a-5p	1	SP1	0
MIMAT0005863	hsa-mir-1200	1	SP1	0
MIMAT0007890	hsa-mir-1914-3p	1	SP1	0
MIMAT0010133	hsa-mir-2110	1	SP1	0
MIMAT0010357	hsa-mir-670-5p	1	SP1	0
MIMAT0012734	hsa-mir-711	1	SP1	0
MIMAT0015002	hsa-mir-466	1	SP1	0
MIMAT0015026	hsa-mir-3153	1	SP1	0
MIMAT0015064	hsa-mir-3184-5p	1	SP1	0
MIMAT0015066	hsa-mir-3065-5p	1	SP1	0
MIMAT0016861	hsa-mir-4308	1	SP1	0
MIMAT0016864	hsa-mir-4312	1	SP1	0

MIMAT0016878	hsa-mir-4257	1	SP1	0
MIMAT0016895	hsa-mir-2355-5p	1	SP1	0
MIMAT0016901	hsa-mir-4271	1	SP1	0
MIMAT0016904	hsa-mir-4276	1	SP1	0
MIMAT0016917	hsa-mir-4287	1	SP1	0
MIMAT0016919	hsa-mir-4292	1	SP1	0
MIMAT0018109	hsa-mir-3681-3p	1	SP1	0
MIMAT0018122	hsa-mir-3692-3p	1	SP1	0
MIMAT0018357	hsa-mir-3941	1	SP1	0
MIMAT0018943	hsa-mir-4428	1	SP1	0
MIMAT0019028	hsa-mir-4493	1	SP1	0
MIMAT0019069	hsa-mir-4530	1	SP1	0
MIMAT0019072	hsa-mir-4533	1	SP1	0
MIMAT0019201	hsa-mir-3127-3p	1	SP1	0
MIMAT0019204	hsa-mir-3140-5p	1	SP1	0
MIMAT0019743	hsa-mir-4667-5p	1	SP1	0
MIMAT0019754	hsa-mir-4672	1	SP1	0
MIMAT0019772	hsa-mir-4685-3p	1	SP1	0
MIMAT0019781	hsa-mir-4691-5p	1	SP1	0
MIMAT0019796	hsa-mir-4700-5p	1	SP1	0
MIMAT0019827	hsa-mir-4716-3p	1	SP1	0
MIMAT0019844	hsa-mir-4725-3p	1	SP1	0
MIMAT0019882	hsa-mir-4747-5p	1	SP1	0
MIMAT0019886	hsa-mir-4749-3p	1	SP1	0
MIMAT0019895	hsa-mir-4755-5p	1	SP1	0
MIMAT0019953	hsa-mir-2467-3p	1	SP1	0
MIMAT0021034	hsa-mir-5006-3p	1	SP1	0
MIMAT0021045	hsa-mir-5011-5p	1	SP1	0
MIMAT0021121	hsa-mir-5190	1	SP1	0
MIMAT0021125	hsa-mir-5194	1	SP1	0
MIMAT0021128	hsa-mir-5196-5p	1	SP1	0
MIMAT0022279	hsa-mir-5582-5p	1	SP1	0
MIMAT0022299	hsa-mir-5590-5p	1	SP1	0
MIMAT0022476	hsa-mir-5692c	1	SP1	0
MIMAT0022497	hsa-mir-5692b	1	SP1	0
MIMAT0022724	hsa-mir-1277-5p	1	SP1	0
MIMAT0022844	hsa-mir-216a-3p	1	SP1	0
MIMAT0023708	hsa-mir-6083	1	SP1	0
MIMAT0024597	hsa-mir-6124	1	SP1	0
MIMAT0024599	hsa-mir-6126	1	SP1	0
MIMAT0026482	hsa-mir-190a-3p	1	SP1	0

MIMAT0026609	hsa-mir-520f-5p	1	SP1	0
MIMAT0026621	hsa-mir-605-3p	1	SP1	0
MIMAT0026623	hsa-mir-627-3p	1	SP1	0
MIMAT0026640	hsa-mir-670-3p	1	SP1	0
MIMAT0026721	hsa-mir-216b-3p	1	SP1	0
MIMAT0027367	hsa-mir-6733-5p	1	SP1	0
MIMAT0027369	hsa-mir-6734-5p	1	SP1	0
MIMAT0027377	hsa-mir-6738-5p	1	SP1	0
MIMAT0027379	hsa-mir-6739-5p	1	SP1	0
MIMAT0027395	hsa-mir-6747-3p	1	SP1	0
MIMAT0027399	hsa-mir-6749-3p	1	SP1	0
MIMAT0027405	hsa-mir-6752-3p	1	SP1	0
MIMAT0027413	hsa-mir-6756-3p	1	SP1	0
MIMAT0027482	hsa-mir-6791-5p	1	SP1	0
MIMAT0027485	hsa-mir-6792-3p	1	SP1	0
MIMAT0027487	hsa-mir-6793-3p	1	SP1	0
MIMAT0027488	hsa-mir-6794-5p	1	SP1	0
MIMAT0027493	hsa-mir-6796-3p	1	SP1	0
MIMAT0027565	hsa-mir-6832-3p	1	SP1	0
MIMAT0027571	hsa-mir-6835-3p	1	SP1	0
MIMAT0027572	hsa-mir-6780b-5p	1	SP1	0
MIMAT0027604	hsa-mir-6852-5p	1	SP1	0
MIMAT0027619	hsa-mir-6859-3p	1	SP1	0
MIMAT0027642	hsa-mir-6871-5p	1	SP1	0
MIMAT0028228	hsa-mir-7159-5p	1	SP1	0
MIMAT0028231	hsa-mir-7160-3p	1	SP1	0
MIMAT0031016	hsa-mir-8089	1	SP1	0
MIMAT0031180	hsa-mir-7977	1	SP1	0
MIMAT0033692	hsa-mir-8485	1	SP1	0
MIMAT0005794	hsa-miR-1296-5p	1	SP1	0
MIMAT0000691	hsa-miR-130b-3p	1	SP1	0
MIMAT0000232	hsa-miR-199a-3p	1	SP1	0
MIMAT0000760	hsa-miR-331-3p	1	SP1	0
MIMAT0003254	hsa-miR-548b-3p	1	SP1	0
MIMAT0000064	hsa-let-7c-5p	1	MYC	0
MIMAT0000065	hsa-let-7d-5p	1	MYC	0
MIMAT0000066	hsa-let-7e-5p	1	MYC	0
MIMAT0000073	hsa-mir-19a-3p	1	MYC	0
MIMAT0000078	hsa-mir-23a-3p	1	MYC	0
MIMAT0000081	hsa-mir-25-3p	1	MYC	0
MIMAT0000082	hsa-mir-26a-5p	1	MYC	0

MIMAT0000086	hsa-mir-29a-3p	1	MYC	0
MIMAT0000088	hsa-mir-30a-3p	1	MYC	0
MIMAT0000096	hsa-mir-98-5p	1	MYC	0
MIMAT0000226	hsa-mir-196a-5p	1	MYC	0
MIMAT0000243	hsa-mir-148a-3p	1	MYC	0
MIMAT0000244	hsa-mir-30c-5p	1	MYC	0
MIMAT0000245	hsa-mir-30d-5p	1	MYC	0
MIMAT0000255	hsa-mir-34a-5p	1	MYC	0
MIMAT0000269	hsa-mir-212-3p	1	MYC	0
MIMAT0000279	hsa-mir-222-3p	1	MYC	0
MIMAT0000414	hsa-let-7g-5p	1	MYC	0
MIMAT0000415	hsa-let-7i-5p	1	MYC	0
MIMAT0000428	hsa-mir-135a-5p	1	MYC	0
MIMAT0000444	hsa-mir-126-5p	1	MYC	0
MIMAT0000454	hsa-mir-184	1	MYC	0
MIMAT0000455	hsa-mir-185-5p	1	MYC	0
MIMAT0000510	hsa-mir-320a	1	MYC	0
MIMAT0000683	hsa-mir-302a-5p	1	MYC	0
MIMAT0000686	hsa-mir-34c-5p	1	MYC	0
MIMAT0000693	hsa-mir-30e-3p	1	MYC	0
MIMAT0000723	hsa-mir-371a-3p	1	MYC	0
MIMAT0000732	hsa-mir-378a-3p	1	MYC	0
MIMAT0000755	hsa-mir-323a-3p	1	MYC	0
MIMAT0000762	hsa-mir-324-3p	1	MYC	0
MIMAT0001080	hsa-mir-196b-5p	1	MYC	0
MIMAT0001541	hsa-mir-449a	1	MYC	0
MIMAT0001631	hsa-mir-451a	1	MYC	0
MIMAT0002822	hsa-mir-512-5p	1	MYC	0
MIMAT0003180	hsa-mir-487b-3p	1	MYC	0
MIMAT0003218	hsa-mir-92b-3p	1	MYC	0
MIMAT0003225	hsa-mir-561-3p	1	MYC	0
MIMAT0003252	hsa-mir-586	1	MYC	0
MIMAT0003267	hsa-mir-599	1	MYC	0
MIMAT0003291	hsa-mir-622	1	MYC	0
MIMAT0003301	hsa-mir-33b-5p	1	MYC	0
MIMAT0004491	hsa-mir-19b-1-5p	1	MYC	0
MIMAT0004492	hsa-mir-19b-2-5p	1	MYC	0
MIMAT0004498	hsa-mir-25-5p	1	MYC	0
MIMAT0004507	hsa-mir-92a-1-5p	1	MYC	0
MIMAT0004508	hsa-mir-92a-2-5p	1	MYC	0
MIMAT0004518	hsa-mir-16-2-3p	1	MYC	0

MIMAT0004549	hsa-mir-148a-5p	1	MYC	0
MIMAT0004551	hsa-mir-30d-3p	1	MYC	0
MIMAT0004602	hsa-mir-125a-3p	1	MYC	0
MIMAT0004605	hsa-mir-129-2-3p	1	MYC	0
MIMAT0004615	hsa-mir-195-3p	1	MYC	0
MIMAT0004676	hsa-mir-34b-3p	1	MYC	0
MIMAT0004689	hsa-mir-377-5p	1	MYC	0
MIMAT0004697	hsa-mir-151a-5p	1	MYC	0
MIMAT0004770	hsa-mir-516a-5p	1	MYC	0
MIMAT0004784	hsa-mir-455-3p	1	MYC	0
MIMAT0004945	hsa-mir-744-5p	1	MYC	0
MIMAT0005789	hsa-mir-513c-5p	1	MYC	0
MIMAT0005792	hsa-mir-320b	1	MYC	0
MIMAT0005884	hsa-mir-1294	1	MYC	0
MIMAT0005892	hsa-mir-1304-5p	1	MYC	0
MIMAT0006767	hsa-mir-1827	1	MYC	0
MIMAT0014998	hsa-mir-3133	1	MYC	0
MIMAT0015031	hsa-mir-3157-5p	1	MYC	0
MIMAT0015033	hsa-mir-3159	1	MYC	0
MIMAT0015087	hsa-mir-514b-5p	1	MYC	0
MIMAT0016867	hsa-mir-4316	1	MYC	0
MIMAT0016920	hsa-mir-4289	1	MYC	0
MIMAT0018193	hsa-mir-3919	1	MYC	0
MIMAT0018941	hsa-mir-4426	1	MYC	0
MIMAT0018948	hsa-mir-4432	1	MYC	0
MIMAT0019047	hsa-mir-4510	1	MYC	0
MIMAT0019709	hsa-mir-4647	1	MYC	0
MIMAT0019736	hsa-mir-4662b	1	MYC	0
MIMAT0019761	hsa-mir-4677-3p	1	MYC	0
MIMAT0019782	hsa-mir-4691-3p	1	MYC	0
MIMAT0019795	hsa-mir-4699-3p	1	MYC	0
MIMAT0019910	hsa-mir-4762-5p	1	MYC	0
MIMAT0019972	hsa-mir-4797-5p	1	MYC	0
MIMAT0021130	hsa-mir-5197-5p	1	MYC	0
MIMAT0022292	hsa-mir-548au-3p	1	MYC	0
MIMAT0023711	hsa-mir-6086	1	MYC	0
MIMAT0025471	hsa-mir-6507-3p	1	MYC	0
MIMAT0026626	hsa-mir-655-5p	1	MYC	0
MIMAT0026719	hsa-mir-889-5p	1	MYC	0
MIMAT0027430	hsa-mir-6765-5p	1	MYC	0
MIMAT0031001	hsa-mir-8074	1	MYC	0

MIMAT0000260	hsa-miR-182-3p	1	MYC	0
MIMAT0000072	hsa-mir-18a-5p	1	HIF1A	0
MIMAT0000103	hsa-mir-106a-5p	1	HIF1A	0
MIMAT0000104	hsa-mir-107	1	HIF1A	0
MIMAT0000263	hsa-mir-199b-5p	1	HIF1A	0
MIMAT0000267	hsa-mir-210-3p	1	HIF1A	0
MIMAT0000274	hsa-mir-217	1	HIF1A	0
MIMAT0000430	hsa-mir-138-5p	1	HIF1A	0
MIMAT0000433	hsa-mir-142-5p	1	HIF1A	0
MIMAT0000757	hsa-mir-151a-3p	1	HIF1A	0
MIMAT0000763	hsa-mir-338-3p	1	HIF1A	0
MIMAT0001341	hsa-mir-424-5p	1	HIF1A	0
MIMAT0001412	hsa-mir-18b-5p	1	HIF1A	0
MIMAT0001413	hsa-mir-20b-5p	1	HIF1A	0
MIMAT0001627	hsa-mir-433-3p	1	HIF1A	0
MIMAT0002832	hsa-mir-519c-3p	1	HIF1A	0
MIMAT0002853	hsa-mir-519d-3p	1	HIF1A	0
MIMAT0002890	hsa-mir-299-5p	1	HIF1A	0
MIMAT0003222	hsa-mir-558	1	HIF1A	0
MIMAT0003256	hsa-mir-589-3p	1	HIF1A	0
MIMAT0004517	hsa-mir-106a-3p	1	HIF1A	0
MIMAT0004607	hsa-mir-138-1-3p	1	HIF1A	0
MIMAT0004657	hsa-mir-200c-5p	1	HIF1A	0
MIMAT0004672	hsa-mir-106b-3p	1	HIF1A	0
MIMAT0004768	hsa-mir-497-3p	1	HIF1A	0
MIMAT0004779	hsa-mir-509-5p	1	HIF1A	0
MIMAT0004796	hsa-mir-576-3p	1	HIF1A	0
MIMAT0004808	hsa-mir-625-3p	1	HIF1A	0
MIMAT0004921	hsa-mir-889-3p	1	HIF1A	0
MIMAT0004975	hsa-mir-509-3-5p	1	HIF1A	0
MIMAT0004978	hsa-mir-935	1	HIF1A	0
MIMAT0005953	hsa-mir-1322	1	HIF1A	0
MIMAT0014983	hsa-mir-3121-3p	1	HIF1A	0
MIMAT0014991	hsa-mir-3128	1	HIF1A	0
MIMAT0015027	hsa-mir-3074-3p	1	HIF1A	0
MIMAT0015034	hsa-mir-3160-3p	1	HIF1A	0
MIMAT0016912	hsa-mir-4282	1	HIF1A	0
MIMAT0017986	hsa-mir-3609	1	HIF1A	0
MIMAT0018083	hsa-mir-3662	1	HIF1A	0
MIMAT0018091	hsa-mir-3668	1	HIF1A	0
MIMAT0018930	hsa-mir-4418	1	HIF1A	0

MIMAT0018972	hsa-mir-548ah-5p	1	HIF1A	0
MIMAT0018987	hsa-mir-4463	1	HIF1A	0
MIMAT0018988	hsa-mir-4464	1	HIF1A	0
MIMAT0018997	hsa-mir-4470	1	HIF1A	0
MIMAT0019021	hsa-mir-4487	1	HIF1A	0
MIMAT0019035	hsa-mir-4499	1	HIF1A	0
MIMAT0019731	hsa-mir-4662a-5p	1	HIF1A	0
MIMAT0019861	hsa-mir-4735-3p	1	HIF1A	0
MIMAT0019884	hsa-mir-4748	1	HIF1A	0
MIMAT0019976	hsa-mir-4799-5p	1	HIF1A	0
MIMAT0021086	hsa-mir-5094	1	HIF1A	0
MIMAT0022480	hsa-mir-5681b	1	HIF1A	0
MIMAT0022484	hsa-mir-5692a	1	HIF1A	0
MIMAT0022984	hsa-mir-5089-3p	1	HIF1A	0
MIMAT0025478	hsa-mir-6511a-5p	1	HIF1A	0
MIMAT0026480	hsa-mir-153-5p	1	HIF1A	0
MIMAT0026555	hsa-mir-329-5p	1	HIF1A	0
MIMAT0026917	hsa-mir-1910-3p	1	HIF1A	0
MIMAT0027410	hsa-mir-6755-5p	1	HIF1A	0
MIMAT0027512	hsa-mir-6806-5p	1	HIF1A	0
MIMAT0027515	hsa-mir-6807-3p	1	HIF1A	0
MIMAT0027516	hsa-mir-6808-5p	1	HIF1A	0
MIMAT0027531	hsa-mir-6815-3p	1	HIF1A	0
MIMAT0027686	hsa-mir-6893-5p	1	HIF1A	0
MIMAT0030982	hsa-mir-8055	1	HIF1A	0
MIMAT0000099	hsa-miR-101-3p	1	HIF1A	0
MIMAT0000071	hsa-miR-17-3p	1	HIF1A	0
MIMAT0002891	hsa-miR-18a-3p	1	HIF1A	0
MIMAT0000278	hsa-miR-221-3p	1	HIF1A	0
MIMAT0000087	hsa-miR-30a-5p	1	HIF1A	0
MIMAT0000773	hsa-miR-346	1	HIF1A	0
MIMAT0019707	hsa-miR-4646-5p	1	HIF1A	0
MIMAT0003316	hsa-miR-646	1	HIF1A	0

Supplemental Table S4. Effect of M_4N on the expression of MYC mRNA in LNCaP prostate, AsPC1 pancreatic, and L428 leukemic cells at 6hrs after treatment of M_4N.

Cell lines	Control	M4N	log2(fold change)
LNCaP	90.97	36.67	-1.31
AsPC1	50.79	32.15	-0.66
L428	65.74	139.69	1.09

LNCaP, AsPC1, and L428 cells were treated with M_4N (60μM) for 6hrs and the amount of MYC mRNA was measured by DNA deep sequencing analyses.

Materials & Methods. Deep RNA sequencing analysis: LNCaP, AsPC-1, and L428 cells were cultured in T75 flasks as described before [13] and treated with M4N (60μM) for 6 h. RNA was extracted from the cells by Trizol reagent (Invitrogen, Carlsbad, CA) and was cleaned by RNeasy kit (Qiagen, Valencia, CA), according to the manufacturer's protocol. The deep RNA sequencing analysis was done at the Deep Sequencing and Microarray Core, Johns Hopkins Medical Institutes (Baltimore, MD). To analyze RNA sequence data, reads were first mapped to human genome (hg19) using TopHat 1.4 [37] and differential expression was detected using the cuffdiff module in Cufflinks [38] and ensembl transcriptome as a guide.
